# Commercial Metallized Textiles for Dry EEG Electrodes: Structure, Textile-Level Durability, and Controlled Frontal Validation Against Ag/AgCl

**DOI:** 10.3390/s26165036

**Published:** 2026-08-08

**Authors:** Eliana Vanesa Andrés Yanzón, Jordi Ferre-Martínez, Daniel López-Rodríguez, Raquel Cervigón, Andrés Camacho-García, Raúl Llinares Llopis

**Affiliations:** 1 Escola Politècnica Superior d’Alcoi, Universitat Politècnica de València, 03801 Alcoy, Spain; evandyan@alumni.upv.es; 2 Asociación de Investigación de la Industria Textil y Cosmética, AITEX Research and Innovation Center, 03802 Alcoy, Spain; jordi.ferre@aitex.es; 3 Departamento de Matemática Aplicada, Universitat Politècnica de València, 03801 Alcoy, Spain; dalorod@upv.es; 4 Polytechnic School, University of Castilla-La Mancha, 16071 Cuenca, Spain; raquel.cervigon@uclm.es; 5 Departamento de Comunicaciones, Universitat Politècnica de València, 03801 Alcoy, Spain; acamacho@dcom.upv.es

**Keywords:** electroencephalography, textile electrodes, dry electrodes, e-textiles, wearable EEG, textile-level laundering durability, electrode–phantom impedance, Ag/AgCl validation

## Abstract

Commercially available metallized textiles offer a scalable material platform for dry EEG electrodes, but their performance depends on the fabric architecture, coating distribution, and durability of the textile component. This work evaluated three industrial conductive textiles—a weft-knit silver-plated PA6/elastane, a warp-knit PA6/elastomer with one-sided conductive silicone, and a woven PA66 with Ag/Cu/Sn metallization—using a standardized, textile-centered protocol combining textile-level washing durability (UNE-EN IEC 63203-204-1:2023), artificial perspiration exposure (UNE-EN ISO 105-E04:2013), impedance characterization on a skin-mimicking phantom with reduced equivalent-circuit analysis, and human EEG recordings (*n* = 9) against a simultaneously recorded Ag/AgCl comparison electrode at Fp1 under controlled resting conditions. The fabric structure and tested surface orientation influenced resistance and textile-level laundering durability, with the woven Ag/Cu/Sn fabric showing the greatest resistance stability across 20 laundering cycles, illustrating a trade-off between conformability and electrical stability. Agreement was quantified using repeated-measures Bland–Altman limits, Lin’s concordance correlation coefficient (CCC), and equivalence testing. The four textile configurations evaluated in the full EEG cohort showed high temporal waveform agreement with the Ag/AgCl comparison signal (grand-mean *r* = 0.92) and a small system-level RMS bias (−7%; CCC = 0.96), with spectral coherence highest in the alpha and lower-frequency bands. No configuration effect was detected for the main agreement metrics within this sample. These findings support commercial metallized textiles as promising dry EEG electrode candidates for further development toward scalable wearable systems.

## 1. Introduction

Electroencephalography (EEG) is a non-invasive technique that records cortical activity with millisecond temporal resolution, and is widely used in clinical diagnosis, cognitive neuroscience, and brain–computer interfaces (BCIs) [1]. Conventional wet Ag/AgCl electrodes provide low-impedance ionic coupling but require skin preparation and gel application; gels progressively dry over time, which raises contact impedance, degrades signal quality, and often causes discomfort or skin irritation that constrains long-term and ambulatory monitoring [2,3,4]. These limitations have motivated gel-free alternatives—dry, quasi-dry, and capacitive electrodes—whose performance depends critically on the electrode–skin interface and on high-input-impedance front ends [5].

Recent dry-vs.-wet comparisons have shown that modern dry electrode EEG can approach or match wet electrodes in key metrics—resting-state spectral power, event-related potentials (e.g., P100, P3b), and machine learning classification—while improving the ease of setup and user preference [6,7]. Emerging printable Ag/AgCl arrays tailored for forehead sites further demonstrate stable open-circuit potentials, low electrode–skin impedance, and high signal quality in mobile scenarios [8], while semi-dry electrodes combine dry-like convenience with improved coupling stability using minimal electrolytes [9]. Electrochemical analyses have clarified that the electrode–skin interface of dry electrodes may include additional charge-transfer resistance and more complex frequency dependence than wet interfaces, underlining the role of interface modeling in amplifier and materials selection [10].

Within this landscape, textile-based dry electrodes (textrodes) are particularly attractive for wearable EEG, offering softness, breathability, conformability, and user comfort [11]. Multilayer, sweat-absorbent designs exploit natural moisture at the skin surface to lower contact impedance and maintain stable recordings on forehead sites [12]. Beyond moisture-mediated coupling, conductive polymer and foam-based dry electrodes—including PEDOT:PSS-coated textiles—have demonstrated good washability, mechanical robustness, and EEG signal quality comparable to commercial electrodes, enabling long-term monitoring with low and stable contact impedance [13,14]. At the materials frontier, MXene-based textiles (e.g., Ti_3_C_2_T_x_) have enabled flexible, breathable electrodes with low impedance and strong mechanical stability, broadening the design space for wearable biopotential sensing [15,16,17].

Additional textile and metal-based strategies broaden the design space. Embroidered textrodes with sandwich architectures improve water retention and reduce contact impedance for sustained biopotential acquisition [18], and washability studies on silver-plated textiles have identified detergent chemistry and mechanical action as the drivers of conductivity loss—actionable knowledge for garment care and lifetime specifications [19]. Comparative analyses across textile electrode types (e.g., stainless-steel fabric vs. copper-nickel vs. silver-plated polyamide) have revealed distinct signal-quality tradeoffs and support data-driven material selection [20]. Other approaches—Ti/TiN multichannel arrays [21], magnetron-sputtered CrSiCN films on glass evaluated as dry ECG electrodes [22], foam- and spring-loaded designs [23], soft PDMS electrodes [24], and garment-embedded systems [25]—have highlighted that contact mechanics and sensor–garment co-design are as decisive as material chemistry. As a forward-looking example relevant to EEG, a fully textile sensor layer has been shown to capture brain activity with comfort advantages, offering open datasets for community benchmarking and accelerating translation [26]. Table 1 summarizes the principal wet, semi-dry, and dry electrode–scalp coupling strategies relevant to EEG, together with their main advantages and limitations.

Mechanical design is as decisive as chemistry in the performance of dry textrodes. Polymer foam-backed electrodes increase geometric conformity, expand the real contact area, and reduce impedance under motion [14,33]. Multipin systems show that normal force at the interface is a governing parameter: an optimal range around 2–3 N simultaneously yields low impedance and good wearer comfort on both hairless and hairy sites [29,30]. Capacitive electrodes using conductive foams can further attenuate motion artifacts while preserving signal fidelity [33]. At the other end of the design spectrum, microneedle arrays bypass the stratum corneum to achieve very low impedances, yet their invasiveness and fabrication complexity limit routine all-day use outside the laboratory [32]. Textile hook electrodes have also demonstrated EEG acquisition through hair without head shaving [31], indicating that direct-contact dry acquisition at hair-bearing scalp sites requires a contact architecture able to traverse the hair barrier.

Despite rapid progress with functional coatings and engineered structures, commercial metallized textiles—for example, silver-plated polyamides or woven Ag/Cu fabrics—remain comparatively under-evaluated for EEG, even though they are widely produced at scale for shielding and medical applications. Existing evaluation frameworks are heterogeneous, as textile-level laundering, perspiration response, surface orientation, interface impedance, and human signal agreement are commonly assessed separately using different electrode geometries, fixation conditions, phantoms, frequency ranges, and outcome metrics, limiting direct comparison across textile electrodes [27,28].

Standardization provides an essential bridge between research prototypes and industrial practice. UNE-EN IEC 63203-204-1:2023 specifies a harmonized, household-like laundering-durability procedure for e-textile products with conductive components, including pre- and post-cycle electrical and visual checks [34]. Complementing this, UNE-EN ISO 105-E04:2013 defines acidic and alkaline artificial perspiration solutions and controlled textile-exposure conditions [35]. In the present study, these standards provide reproducible textile-level conditions for evaluating the laundering durability and descriptive resistance responses under artificial perspiration. Resistance and microscopy capture the outcomes of aging—loss of electrical continuity and visible damage to the conductive layer—rather than the electrochemical mechanisms behind it; coating oxidation, corrosion kinetics, polarization behavior, and charge-transfer processes require dedicated electrochemical characterization.

In textile-based electrode development, fabric architecture plays a decisive role alongside chemical composition: elasticity, compressibility, surface topology, and coating symmetry govern electrode–skin coupling, long-term stability, and susceptibility to motion artifacts. Knitted structures conform well to curved surfaces and maintain stable contact during movement, whereas woven fabrics offer higher dimensional stability at the cost of compliance; surface coatings, in turn, may be symmetrically or asymmetrically distributed, further shaping electrode behavior [28].

This work evaluates three commercial Shieldex^®^ textiles as dry EEG electrode candidates, which were deliberately chosen to span different architectures rather than a single construction: (i) a weft-knit polyamide 6/elastane (PA6/EL) with bare silver plating, (ii) a warp-knit PA6/elastomer with one-sided conductive silicone, and (iii) a woven PA66 with Ag/Cu/Sn metallization. Treating surface orientation as an explicit design variable for the two fabrics with an a priori face/reverse contrast, the protocol integrates (a) textile-level laundering durability according to UNE-EN IEC 63203-204-1:2023, with SEM inspection; (b) artificial perspiration exposure at pH 5.5 and 8.0 according to UNE-EN ISO 105-E04:2013, complemented by a neutral demineralized-water control (pH ≈ 7.0); (c) controlled electrode–phantom impedance with reduced equivalent-circuit analysis; and (d) human EEG comparison with a commercial pre-gelled Ag/AgCl comparison electrode at Fp1 under resting conditions, using temporal waveform agreement, amplitude agreement, spectral coherence, and spectral-complexity metrics. A recent study assessed several Shieldex^®^ fabrics for ECG monitoring and reported the best aggregate performance for the conductive-silicone stretch-knit fabric—one of the materials also examined here—although the differences between the materials were not statistically significant [36]. This work, however, addressed electrocardiography only and applied neither standardized laundering nor perspiration protocols, leaving the behavior of these textiles under EEG conditions and textile-realistic stressors unexplored. The scientific necessity and novelty of the present study lie in integrating these normally separate textile-level durability, controlled-interface, and EEG evaluations within one matched workflow for commercially available metallized fabrics. Accordingly, the present work is framed as a first qualification stage. The prototypes carry a planar textile contact surface with no hooks, pins, or comb structures intended to traverse hair, and are therefore evaluated at a hairless frontal site. Complete-device washability, performance on hair-bearing scalp, and robustness to movement remain to be established in subsequent work.

## 2. Materials and Methods

### 2.1. Conductive Fabrics

Three commercially available conductive textiles manufactured and supplied by Statex Produktions- und Vertriebs GmbH (Bremen, Germany) were selected to span distinct textile architectures and coating strategies (Table 2) [37,38,39]. Fabric 1 (Shieldex^®^ Med-tex P180) is a silver-plated PA6/elastane weft knit with a mirror-satin face, whose elasticity favors conformal scalp contact. Fabric 2 (Shieldex^®^ Silitex) is a PA6/elastomer warp knit with a conductive-silicone coating applied only to the face side, creating a smooth conductive face and a softer knitted reverse. Fabric 3 (Shieldex^®^ Zell RS) is a dimensionally stable woven PA66 with Ag/Cu/Sn metallization and minimal stretch. Because surface orientation may influence contact mechanics and electrode–skin coupling, both orientations were evaluated for Fabrics 1 and 2, as each presented an a priori face/reverse contrast: Fabric 1 had a mirror-satin face and a more open knitted reverse, whereas Fabric 2 had a conductive-silicone coating applied only to one side. These configurations were designated F1F/F1R and F2F/F2R, where F and R denote the tested face and reverse orientations, respectively. Fabric 3 was evaluated in one orientation because its woven ripstop construction is metallized with Ag/Cu/Sn and the manufacturer did not identify a one-sided functional coating or a preferred skin-contact face [39]. The designation F3F is retained for nomenclature consistency and identifies the tested orientation; the exact equivalence between the two physical sides was not assessed. Together, these materials span elasticity, compressibility, surface morphology, and coating-symmetry contrasts relevant to dry EEG electrode designs.

### 2.2. Physical and Thermal Characterization

Fiber classification followed UNE 40449:1982 [40], where identification combined stereomicroscopy using an S APO stereomicroscope 10×–50× from Leica (Wetzlar, Germany) to document fiber morphology and coating distribution, with triplicate pyrognostic testing to support polymer identification and differentiation of PA6-, PA66-, and elastomer-containing blends from their combustion behavior.

Polymer composition and thermal stability were further assessed by differential scanning calorimetry (DSC) using a DSC 1 STAR System from Mettler Toledo (Gießen, Germany), analyzing three samples per fabric (2–3 mg) from 30 to 350 °C at 10 °C·min^−1^ under nitrogen. This range covered the melting profiles of PA6 (~220 °C) and PA66 (~260 °C); the melting temperatures were determined by the onset method using Mettler STARe software version 18.0.

### 2.3. Durability Testing

Washing durability was evaluated according to UNE-EN IEC 63203-204-1:2023 [34]. Textile specimens (40 mm × 100 mm; *n* = 3 per fabric) were subjected to 20 domestic washing cycles using washing procedure 9B of UNE-EN ISO 6330:2012 [41] (Type B reference machine, 16 ± 3 °C, normal agitation), with reference detergent but without optical brighteners. After each cycle, specimens were dried flat at room temperature for 8 h (drying procedure C). Electrical resistance was measured before washing and after cycles 1, 5, 10, 15, and 20 using the calibrated digital multimeter Fluke 8845A from Fluke (Everett, WA, USA) in a four-wire configuration. Measurements were taken over a 20 mm separation at three predefined positions along each specimen, with three repeated readings per position. At each time point, the nine technical readings from each specimen were averaged to obtain one specimen-level value; the reported mean and standard deviation were then calculated across the three independent specimens. The face and reverse sides were measured separately for the knitted fabrics (Fabrics 1 and 2), whereas the woven Fabric 3 was measured in the orientation designated F3F. These laundering tests were performed on constituent textile specimens rather than on the complete multilayer electrodes containing conductive foam, seams, and snap connectors; consequently, they assess textile-level electrical durability rather than complete-device washability.

Surface morphology and coating integrity were examined by scanning electron microscopy (SEM) with a TM3000 tabletop microscope from Hitachi (Tokyo, Japan) operating at 15 kV after 10 and 20 washing cycles. Micrographs were acquired at 100×–1000× to assess fiber-level deformation, continuity of the metallic or conductive coatings, cracking, delamination, and other wash-induced degradation patterns. SEM was used as a complementary structural assessment to the resistance measurements, in line with the deterioration-monitoring recommendations of UNE-EN IEC 63203-204-1:2023.

### 2.4. Electrode Design

**Figure 1:**
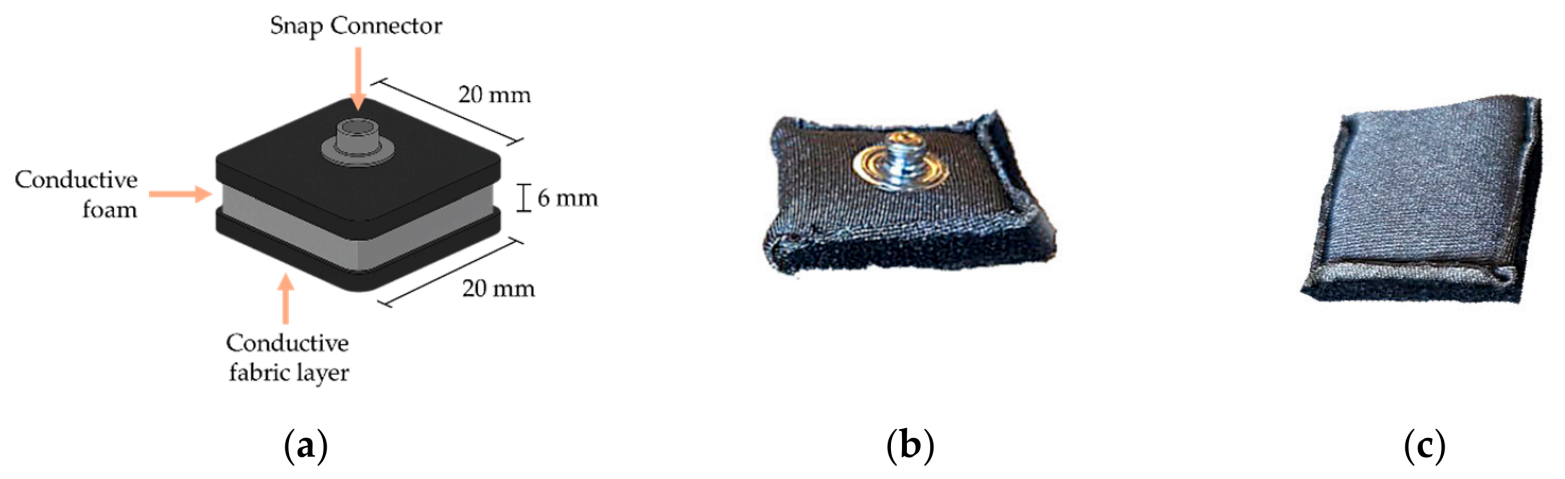
Textile EEG electrode prototype. (**a**) Multilayer structure with the conductive textile layer enclosing a 6 mm conductive foam core and an integrated snap connector. (**b**) Outer side with snap connector. (**c**) Skin-facing textile surface.

Textile EEG electrodes were fabricated as square multilayer prototypes measuring 20 mm × 20 mm, with a total thickness of approximately 7 mm (Figure 1a). A 6 mm conductive polyurethane foam core (RS PRO ESD Foam from RS Components Ltd. (Corby, UK), Stock No. 815-3509; density 35–55 kg·m^−3^; tensile strength 70 kPa; surface resistivity 10^2^–10^5^ Ω/sq; volume resistance < 2.5 × 10^2^ Ω) was wrapped in a single piece of the selected conductive textile, folded over the foam, and then closed along its three open edges with standard non-conductive sewing thread (Figure 1b). The foam provided a compliant conductive backing and mechanical cushioning, while a metal snap connector was embedded on the outer side (Figure 1c), leaving a continuous textile-only surface for skin contact. For each prototype, the textile was folded so that the intended face or reverse surface was exposed on the skin-contact side.

Five surface-specific prototypes were produced to assess fabric architecture and face/reverse orientation: F1F and F1R from Fabric 1, F2F and F2R from Fabric 2, and F3F from Fabric 3. All prototypes shared the same geometry, foam core, and connector layout, differing only in the textile surface exposed to the skin. All five configurations were included in the material and electrode–phantom characterization. Before the full *n* = 9 campaign, F2F was evaluated in a separate four-participant feasibility pilot using the same OpenBCI Cyton acquisition system as the main study but was not carried forward to the full cohort. That decision was informed by its low temporal and spectral agreement in the pilot and its markedly different electrode–phantom response; no prespecified numerical carry-forward criterion had been defined. The pilot is therefore reported as exploratory in Section 3.5 and is not pooled with the full-cohort analysis. The full-cohort EEG comparison was restricted to F1F, F1R, F2R, and F3F, and no participants or observations from the *n* = 9 cohort were excluded on the basis of the recorded outcomes.

### 2.5. Electrical Characterization

Electrical characterization comprised artificial perspiration exposure of constituent textile specimens and impedance characterization on a skin-mimicking phantom. Artificial sweat testing used the acidic (pH ≈ 5.5) and alkaline (pH ≈ 8.0) perspiration solutions specified in UNE-EN ISO 105-E04:2013 [35], together with a neutral demineralized-water control (pH ≈ 7.0). For each fabric surface orientation, three rectangular specimens of the 40 mm × 100 mm size specified in that standard were assigned to acidic, alkaline, or neutral exposure, yielding 15 specimens in total. Thus, each fabric–surface–solution combination was represented by one independent specimen. The nine readings per specimen—three predefined positions × three repeated readings—were technical replicates from that specimen, and the associated standard deviation describes within-specimen measurement repeatability rather than specimen-to-specimen variability. This differs from the laundering experiment, which used *n* = 3 independent specimens per fabric. No inferential comparison between the perspiration conditions was performed, and the results are reported only as preliminary descriptive measurements. Dry resistance was measured on each specimen as a baseline using the four-wire protocol described for washing durability. Specimens were then immersed in the corresponding solution for 30 min at room temperature, gently drained on filter paper, and re-measured while moist in the applicable tested orientation to quantify perspiration-associated resistance changes. The artificial perspiration formulations specified in UNE-EN ISO 105-E04:2013 contained L-histidine monohydrochloride monohydrate (0.5 g/L) and sodium chloride (5.0 g/L), with sodium dihydrogen phosphate dihydrate (2.2 g/L) for the acidic solution and disodium hydrogen phosphate dodecahydrate (5.0 g/L) for the alkaline solution; the pH was adjusted with 0.1 mol·L^−1^ NaOH as specified by the standard.

Electrode–phantom impedance was measured with a B&K Precision 895 LCR meter (Yorba Linda, CA, USA) using a gelatin-based skin phantom adapted from previous EEG phantom studies [42]. The phantom consisted of porcine gelatin (240 Bloom, 16.9 wt%) cast in a 3D-printed container (15 cm × 11 cm), forming a 2.1 cm thick hydrogel slab with an embedded Ag/AgCl electrode positioned centrally 7 mm from the bottom. Textile electrode prototypes and a commercial Ag/AgCl comparison electrode were placed on the phantom surface and connected to the LCR meter using their respective connectors. A 225 g mass was applied to standardize the applied load. Impedance magnitude and phase were acquired from 20 to 100 Hz using a 50 mV test signal, 100 frequency points, a 1 m cable length setting, medium measurement speed, and three internal repetitions per frequency point. The 20 Hz lower bound corresponded to the minimum test frequency of the instrument, whereas 100 Hz was the upper bound selected in the original EEG-oriented protocol. Measurements were performed in a two-electrode configuration between the embedded phantom electrode and the surface device under test, so differences between electrode types reflect contact behavior under the fixed phantom geometry, cabling, and loading conditions.

The raw complex-impedance results were analyzed using the real and imaginary components recorded by the LCR meter. Each complete technical sweep comprised 100 linearly spaced frequencies from 20 to 100 Hz. Only complete 100-point sweeps were retained; partial leading or trailing blocks generated during file export were discarded. Sixteen complete sweeps were retained for each electrode. These were technical repetitions acquired without changing the electrode–phantom placement and were therefore summarized descriptively by medians and interquartile ranges rather than being treated as independent specimens. Accordingly, the reported interquartile ranges characterize within-placement technical repeatability—including any slow drift of the electrode–phantom interface over the course of a measurement session—and do not estimate variability between electrode specimens, independent placements, or repeated donning.

Equivalent-circuit selection was constrained by the restricted frequency range. Three candidate descriptions were evaluated: an ideal series-RC model [43]; the full textile-interface topology $R_{s}+(R_{p}∥CPE)$ reported in the previous literature [44], in which the ideal capacitance is replaced by a constant-phase element (CPE) to represent the distributed, non-ideal response produced by heterogeneous contact; and the reduced $R_{s}$–CPE model:

(1) $$Z\left(f\right)=R_{s}+\frac{1}{Q(j2\pi f{)}^{\alpha }},\;0<\alpha \leq 1$$

Here, $R_{s}$ is an effective series contribution. Given the two-electrode measurement configuration described above, $R_{s}$ is a setup-dependent effective series term that can include contributions from the surface device being tested, the gelatin bulk path, the embedded electrode, the cabling, and the current-distribution geometry; it is therefore not an interface-specific material resistance. Q is the CPE coefficient, and α describes non-ideal frequency dispersion; Q has units of ${S\cdot s}^{\alpha }$ and should not be interpreted as a conventional capacitance unless α=1. Because these units depend on α, numerical Q values should not be compared directly across electrodes with different α.

Positive parameters were optimized in transformed parameter space using multistart nonlinear least squares. At each frequency, real and imaginary residuals were normalized by the measured impedance magnitude and fitted simultaneously. Model comparison used complex normalized root-mean-square error (NRMSE), the Bayesian information criterion (BIC), Jacobian conditioning, and boundary behaviors. For BIC calculation, N=200 scalar residual components were used, corresponding to the real and imaginary residuals at 100 frequencies. For comparison purposes, $\Delta BIC_{model-reduced}$ was defined as $BIC_{model}-BIC_{reduced}$; positive values therefore favor the reduced $R_{s}$–CPE model. Because the fitted parameters were to be compared across electrodes, a single common topology was required. Among the three candidates, the most parsimonious common model that provided an acceptable fit quality across all electrodes within the measured band was retained and applied uniformly; the corresponding model-comparison diagnostics are reported in Section 3.4.

Reduced-model parameters from the complete technical sweeps are reported as medians and interquartile ranges. Within the measured 20–100 Hz interval, the fitted elements provide a compact and reproducible description of how each electrode couples to the phantom. Assigning them to charge-transfer resistance, double-layer capacitance, or oxidation kinetics—or transferring them to the in vivo electrode–skin interface—would require a broader frequency range and a dedicated electrochemical protocol.

### 2.6. EEG Signal Acquisition

A prospective controlled EEG study was conducted with nine healthy volunteers (five males and four females, 25 ± 3 years) under resting laboratory conditions, with minimal acoustic stimulation and no direct lighting. This nine-participant cohort was used as an initial controlled human EEG validation, employing a within-participant paired design, with each participant contributing matched textile–Ag/AgCl recordings for all four full-cohort configurations. All procedures were approved by the Research Ethics Committee of the Universitat Politècnica de València, Spain (protocol P20_31-03-2026).

**Figure 2:**
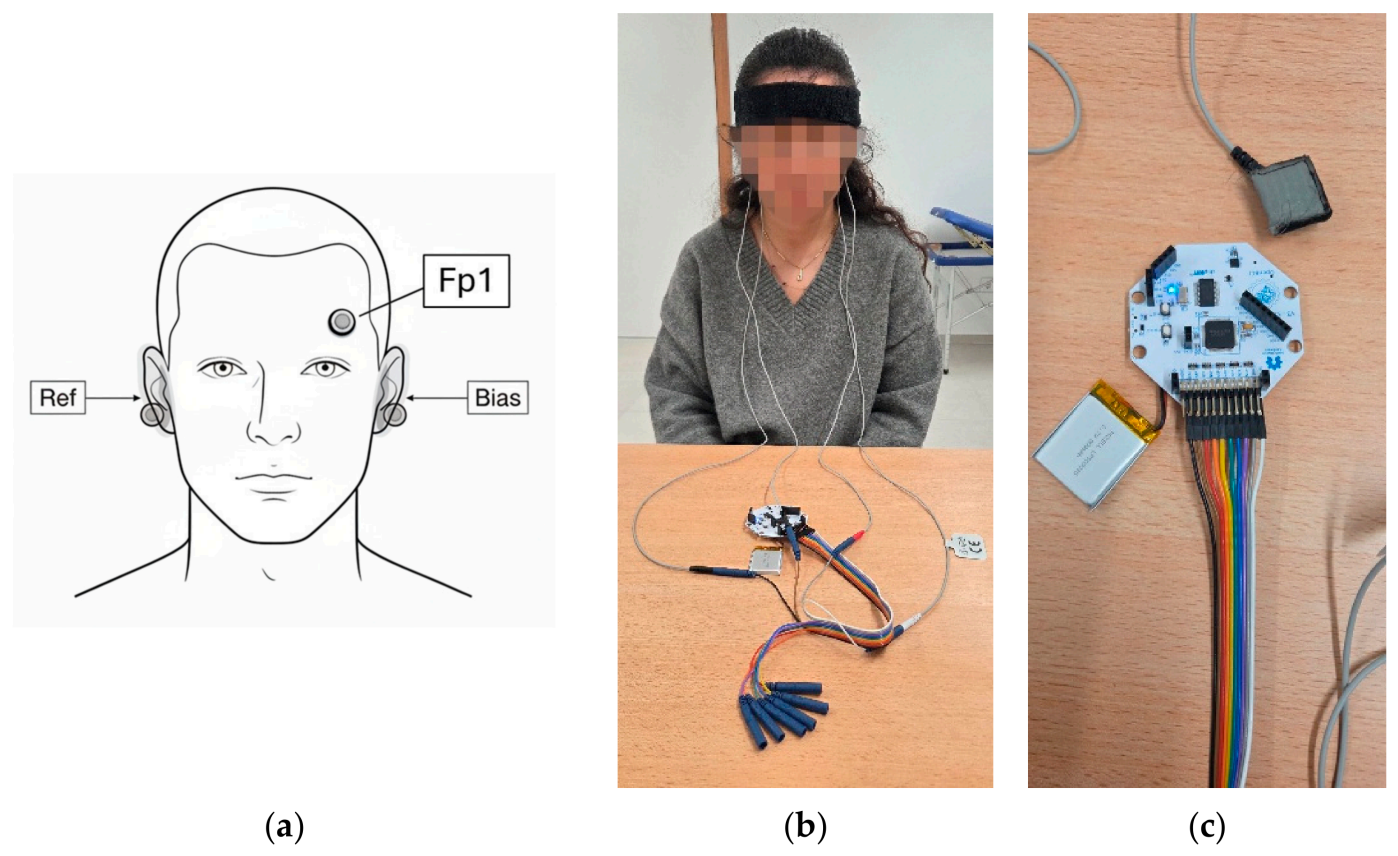
EEG acquisition setup. (**a**) Fp1 recording location according to the international 10–20 system. (**b**) Textile and adjacent Ag/AgCl comparison electrodes placed on the forehead, with REF and BIAS/DRL electrodes on the mastoids. (**c**) OpenBCI Cyton acquisition board used for recording.

Each participant completed an approximately 20 min measurement sequence. The four textile configurations included in the full-cohort analysis were evaluated in a participant-specific randomized order to distribute potential recording-period effects across configurations. Textile and Ag/AgCl comparison electrodes were recorded simultaneously at the left frontal Fp1 region according to the international 10–20 system (Figure 2a). After positioning each textile prototype, a 1 min signal-quality and contact-stability check was performed and excluded from analysis; no separate washout interval was used. A 4 min recording was then acquired, with alternating eyes-open (EO) and eyes-closed (EC) periods every 60 s. Only the textile prototype was exchanged between configurations. The adjacent Fp1 Ag/AgCl comparison electrode and the common REF and BIAS/DRL electrodes remained in place throughout the session.

The Fp1 comparison electrode, the common reference electrode (REF), and the bias/driven-right-leg electrode (BIAS/DRL) were commercial pre-gelled (solid-hydrogel) Ag/AgCl electrodes of the same type, applied as supplied without additional conductive gel or skin abrasion. The Fp1 electrode served as the simultaneously recorded Ag/AgCl comparison electrode. The Ag/AgCl comparison electrode was positioned above the left eyebrow, with the textile electrode placed immediately adjacent to it to enable within-subject, sample-synchronous comparison. REF and BIAS/DRL were placed on the right and left mastoids, respectively.

Each electrode was held in place by its own means of fixation: the pre-gelled Ag/AgCl electrode adhered to the skin through its integrated adhesive, whereas the dry textile electrode was secured with a standard elastic headband (Figure 2b). Contact force was not instrumented or matched between the two electrode types. In addition, the two sensing surfaces were adjacent rather than exactly co-located. Therefore, the paired comparison represents the performance of the complete recording configurations under controlled simultaneous acquisition, but it cannot isolate intrinsic textile-related signal loss from the differences in local position, pressure distribution, attachment, or interface mechanics.

Signals were acquired with an OpenBCI Cyton board (OpenBCI, Brooklyn, NY, USA) (Figure 2c) based on the ADS1299 front end (Texas Instruments, Dallas, TX, USA; input impedance 1000 MΩ), using shielded leads and the OpenBCI GUI for real-time visualization and data capture. The sampling frequency was 250 Hz.

### 2.7. EEG Signal Evaluation

EEG signals recorded simultaneously from textile and Ag/AgCl comparison electrodes were processed with a unified pipeline in MATLAB R2024b (MathWorks, Natick, MA, USA), applying identical parameters to all recordings. The pipeline produced subject-level metrics for each condition (eyes-open, EO; eyes-closed, EC) and textile configuration (F1F, F1R, F2R, and F3F), using the Ag/AgCl comparison signal as the comparator.

Signals were filtered using fixed zero-phase IIR filters, with a fourth-order notch at 50 Hz for power-line interference, followed by a fourth-order band-pass with −3 dB cutoffs at approximately 1 Hz and 50 Hz. Both were applied bidirectionally (forward–reverse, filtfilt) to avoid phase distortion. The first 4.0 s and last 1.0 s of each recording were discarded to remove recording and filter transients. Derived spectral metrics were computed over an effective range of ~1–45 Hz, with the first included spectral bin at 1.25 Hz.

Eye-blink artifacts were detected on the Ag/AgCl comparison channel within eyes-open blocks. Candidate blinks were identified on the smoothed absolute-value trace using an amplitude threshold at Fp1 and a minimum inter-peak separation of 0.8 s, at three thresholds (100, 150, 200 µV) for robustness; at Fp1, blinks reach several hundred microvolts and are well-separated from background EEG. Segments of ±500 ms around each blink were excluded from the brain-activity analyses (temporal correlation, PSD, coherence, entropy) and retained only for blink-morphology analysis.

Temporal similarity was quantified with the Pearson correlation coefficient (*r*) on artifact-free segments within each condition; signals were time-aligned sample-by-sample (shared acquisition clock). Because *r* is bounded and non-normal, values were Fisher-*z*-transformed (*z* = atanh(*r*)) for inference, with descriptive statistics on the original *r* scale. The subject was the independent unit (*n* = 9). Fisher-*z* correlations were analyzed with a linear mixed-effects model (condition × configuration fixed effects, per-subject random intercept, Satterthwaite degrees of freedom), with a 2 × 4 repeated-measures ANOVA as a sensitivity analysis.

Signal amplitude was summarized as the RMS of the filtered EEG, reported as the percent difference relative to the Ag/AgCl comparison signal:

(2) $$\Delta RMS(\%)=100\times ({RMS}_{\mathrm{textile}}-{RMS}_{\mathrm{Ag}/\mathrm{AgCl}})/{RMS}_{\mathrm{Ag}/\mathrm{AgCl}}$$

The RMS log-ratio, log(RMS_textile_/RMS_Ag/AgCl_), was analyzed with the same mixed-effects design. Agreement with the Ag/AgCl comparison signal was assessed by repeated-measures Bland–Altman analysis [45] (limits from the mixed-model variance components), Lin’s concordance correlation coefficient (CCC) [46], and two one-sided tests (TOST) [47] for equivalence of the mean bias, at exploratory margins of ±10%, ±15%, and ±20%. The within-subject association between RMS attenuation and temporal fidelity was assessed with repeated-measures correlation ($r_{rm}$) [48], which removes between-subject variance before estimating the within-subject relationship.

Blink-morphology agreement was assessed from the Pearson correlation between the textile and Ag/AgCl comparison signals within each ±500 ms blink window. Per the subject and configuration, event correlations were averaged in the Fisher-*z* domain (back-transformed for reporting), and configurations compared with the Friedman test on subject-level values (with a mixed-effects model as a parametric check), restricted to eyes-open blocks and repeated at the three thresholds (100, 150, 200 µV).

Spectral similarity was quantified as magnitude-squared coherence (Welch’s method, 2 s Hann windows, 50% overlap, 1–45 Hz), computed per clean interval and aggregated by weighting component spectra by their number of Welch windows. Coherence was band-averaged within delta (1–4 Hz), theta (4–8 Hz), alpha (8–13 Hz), and beta (13–30 Hz), logit-transformed (clipped to [0.001, 0.999]) for inference, with descriptive statistics on the original scale (subject as independent unit, *n* = 9). Logit-coherence was analyzed with a linear mixed-effects model (condition × band × configuration fixed effects, per-subject random intercept, Satterthwaite degrees of freedom), with a Greenhouse–Geisser-corrected repeated-measures ANOVA as a sensitivity analysis.

Power spectral density (PSD) was estimated with Welch’s method (2 s Hann windows, 50% overlap, 1000-point FFT; 0.25 Hz resolution). Spectral entropy was computed from the normalized PSD over 1–45 Hz as Shannon entropy divided by log_2_(*N*), where *N* is the number of spectral points, yielding values in [0, 1] (effective lower bin 1.25 Hz). Entropy was logit-transformed and analyzed with a mixed-effects model (configuration × condition, per-subject random intercept). As a secondary descriptive measure, alpha reactivity was defined per subject and configuration as the eyes-closed/eyes-open alpha-power ratio (8–13 Hz) and compared between electrode types. For the derived amplitude and complexity features (RMS and spectral entropy), textile–Ag/AgCl agreement was quantified on subject-level paired differences using repeated-measures Bland–Altman analysis (limits from mixed-model variance components), Lin’s CCC, and TOST; where a significant proportional bias was detected, magnitude-dependent limits of agreement were reported instead of constant limits.

Across all metrics, statistical significance was set at α = 0.05 (two-tailed), and multiple comparisons were corrected with the Holm procedure within predefined families of tests, excluding intercept terms. Given the sample size (*n* = 9), subject-level cluster-bootstrap confidence intervals were computed for the concordance coefficients, with degenerate intervals flagged. Table 3 summarizes the EEG metrics, their statistical transformations, and the inferential test applied to each.

## 3. Results and Discussion

### 3.1. Physical and Thermal Characterization

**Figure 3:**
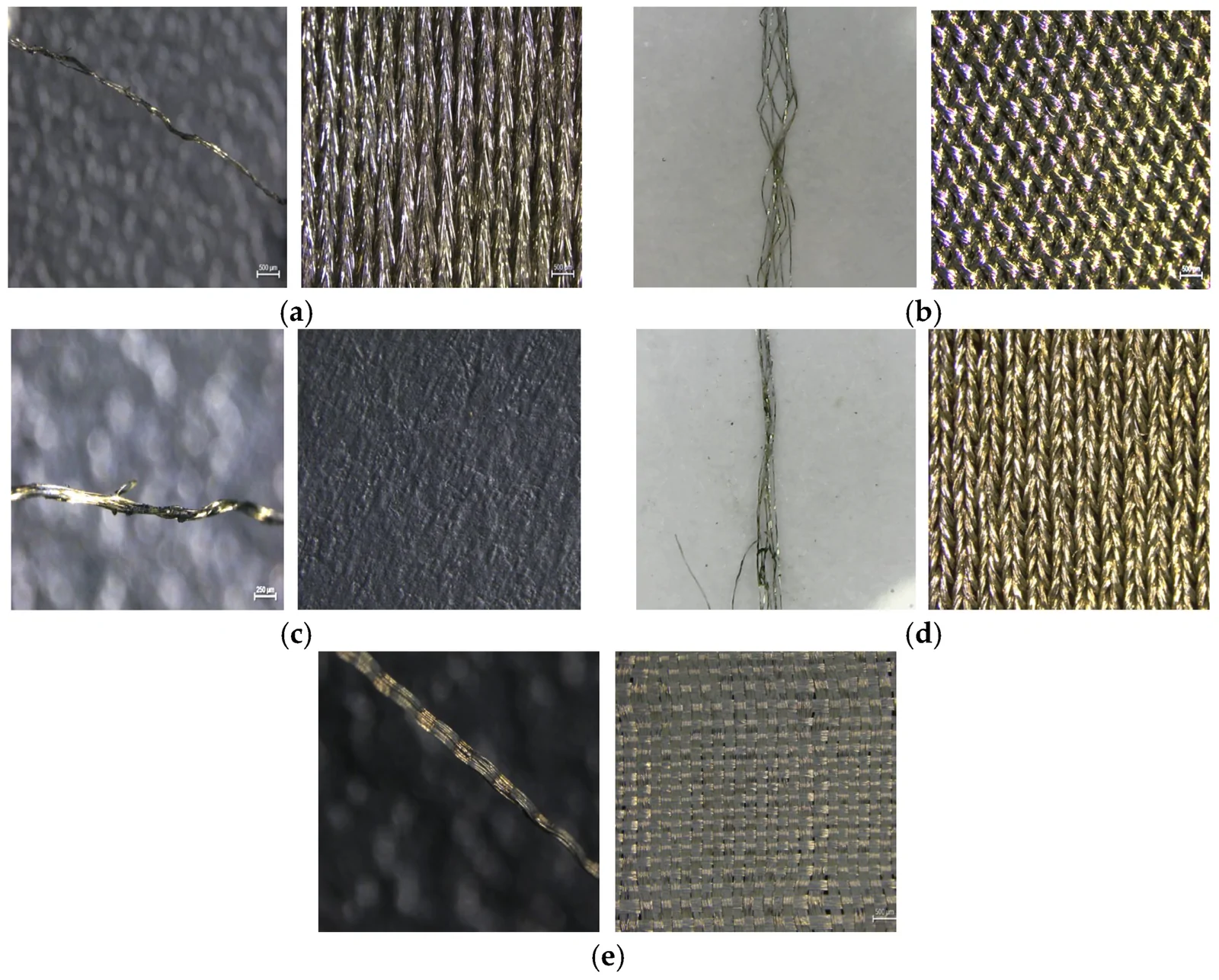
Stereomicroscopic images of the three conductive textiles used for electrode fabrication. For each fabric, individual filaments and the corresponding fabric structure are shown. (**a**) Fabric 1, silver-plated PA6 filament and weft-knit face (mirror-satin). (**b**) Fabric 1—fiber bundle and weft-knit reverse (open looped structure). (**c**) Fabric 2—silver-plated PA6/elastomer construction and silicone-coated face. (**d**) Fabric 2—fiber bundle and warp-knit stretch-tricot reverse. (**e**) Fabric 3—PA66 filament with Ag/Cu/Sn metallization and woven ripstop structure.

Microscopic and thermal characterization confirmed that the three commercial textiles represented distinct material and structural profiles relevant to dry EEG electrode designs. Stereomicroscopy showed that Fabric 1 was a compliant silver-plated PA6/elastane weft knit with a smoother mirror-satin face and a more open looped reverse, whereas Fabric 2 was a warp-knit PA6/elastomer textile with a continuous conductive-silicone coating on the face side and an exposed knitted reverse. Fabric 3 differed markedly from both knitted structures, showing a dense woven PA66 ripstop architecture with Ag/Cu/Sn metallization distributed along the yarns (Figure 3). These observations establish the main design contrast of the study: the knitted fabrics provide greater compliance and surface adaptability, whereas the woven fabric provides higher dimensional stability but lower compressibility.

Pyrognostic testing supported the polyamide-based composition of the three fabrics. Fabrics 1 and 2 showed melting and bead formation consistent with PA6-based materials, while Fabric 3 burned more slowly and produced a gray ash pattern consistent with PA66. These observations were consistent with the manufacturer-declared polymer compositions and with the different mechanical behavior observed under microscopy.

**Figure 4:**
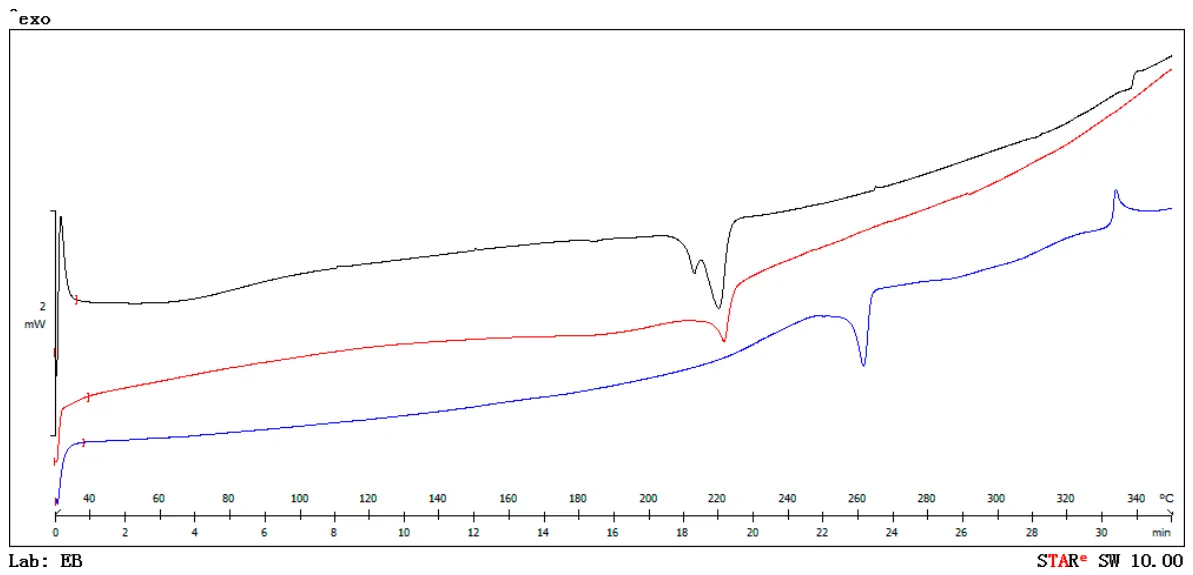
Differential scanning calorimetry thermograms of the three conductive textiles. Fabric 1 (red) showed a PA6 melting peak at approximately 223 °C, Fabric 2 (black) showed two PA6-related melting events at approximately 211 °C and 223 °C, and Fabric 3 (blue) showed a PA66 melting peak at approximately 261 °C.

Differential scanning calorimetry further differentiated the textile substrates (Figure 4). Fabric 1 showed a single melting peak at approximately 223 °C, characteristic of PA6. Fabric 2 showed two melting events at approximately 211 °C and 223 °C, consistent with PA6-based components with different thermal histories or crystalline populations; the elastomeric component was not resolved as a separate endothermic transition. Fabric 3 showed a sharper melting peak at approximately 261 °C, consistent with PA66 and its higher dimensional stability. All melting transitions were far above any temperature reached during the domestic laundering protocol, indicating that subsequent wash-induced changes were more likely related to coating integrity, yarn structure, and conductive-path disruption than to bulk polymer thermal instability.

### 3.2. Textile-Level Washing Durability

**Figure 5:**
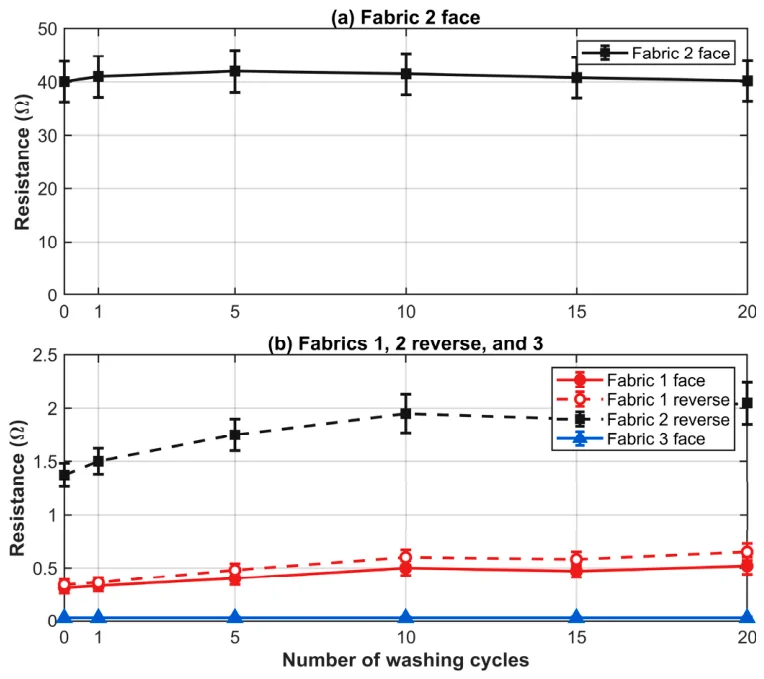
Washing-induced resistance changes over 20 cycles. Error bars indicate standard deviation across *n* = 3 specimens. The silicone-coated face of Fabric 2 is shown on a separate scale because its absolute resistance was markedly higher than that of the other textile surfaces.

Electrical resistance was monitored over twenty washing cycles to assess the stability of the conductive pathways under standardized laundering conditions (Table 4; Figure 5). All fabrics remained conductive after the complete washing protocol, but their resistance trajectories differed substantially according to textile architecture and surface treatment. Fabric 3, the woven PA66 textile with Ag/Cu/Sn metallization, showed the greatest electrical stability across 20 laundering cycles: the face-side resistance remained very low and practically unchanged, with mean values close to 0.03 Ω throughout the 20 cycles. Fabric 1, the silver-plated PA6/elastane weft knit, remained within a low-ohmic range but showed a progressive resistance increase, from 0.31 ± 0.05 Ω to 0.52 ± 0.08 Ω on the face side and from 0.34 ± 0.05 Ω to 0.65 ± 0.08 Ω on the reverse side, after 20 cycles. Fabric 2 showed the strongest surface-dependent behavior: the silicone-coated face remained comparatively stable around 40 Ω but at a much higher absolute resistance, whereas the uncoated reverse increased from 1.37 ± 0.11 Ω to 2.05 ± 0.20 Ω. Thus, textile-level laundering durability was governed by both resistance stability and absolute resistance levels rather than by a single monotonic ranking across the fabrics.

These resistance trajectories describe the constituent textile specimens under the standardized 20-cycle protocol. Extending them to the assembled electrode would additionally require testing the foam integrity, seams, connector fixation, and interlayer electrical continuity.

**Figure 6:**
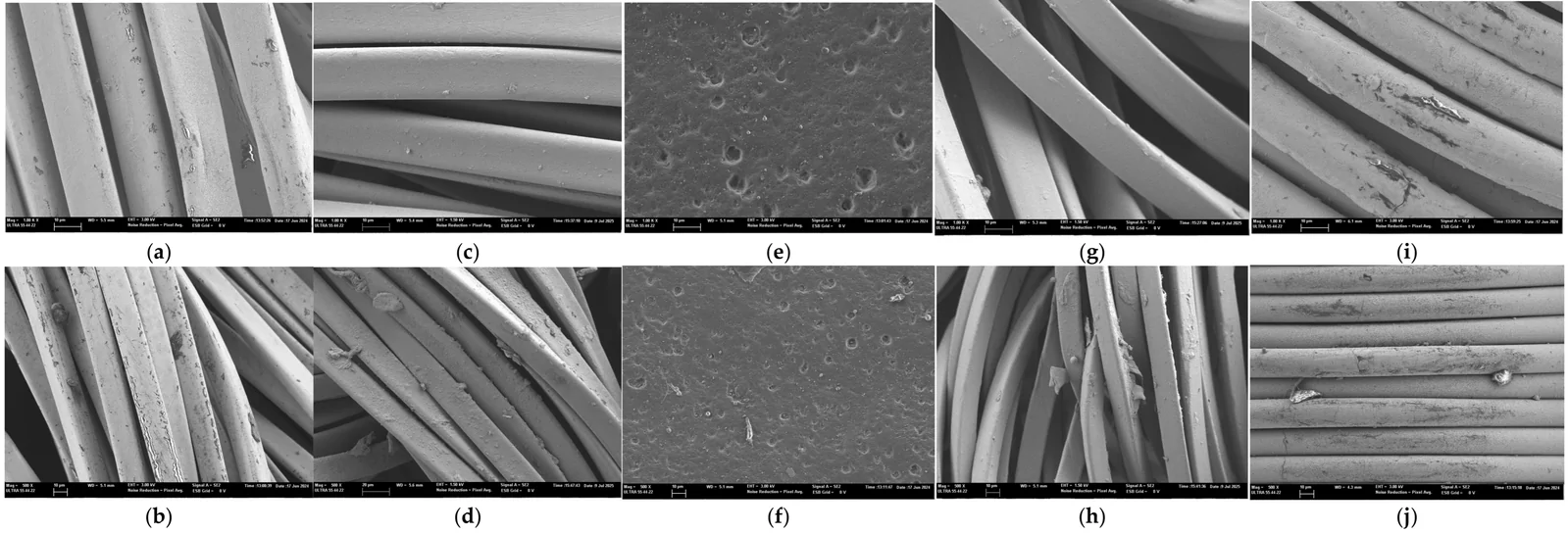
Scanning electron microscopy (SEM) images of conductive fabrics after two different washing cycles. Specimens after 10 cycles were imaged at 1000× and specimens after 20 cycles at 500× (scale bars shown in each micrograph). Fabric 1: Face after 10 cycles (**a**) and face after 20 cycles (**b**). Fabric 1: Reverse after 10 cycles (**c**) and reverse after 20 cycles (**d**). Fabric 2: Face after 10 cycles (**e**) and face after 20 cycles (**f**). Fabric 2: Reverse after 10 cycles (**g**) and reverse after 20 cycles (**h**). Fabric 3: Tested F3F orientation after 10 cycles (**i**) and after 20 cycles (**j**).

SEM observations after repeated laundering supported the electrical measurements and clarified the morphology associated with different resistance trajectories (Figure 6). Fabric 1 showed visible coating deterioration after washing, especially on the reverse side, with cracking, thinning, and partial detachment of the silver layer, consistent with the progressive resistance increase observed electrically. With Fabric 2, the silicone-coated face remained comparatively continuous after washing, whereas the exposed knitted reverse showed localized rupture and separation marks. Fabric 3 exhibited minimal structural degradation, with the woven yarns and Ag/Cu/Sn coating largely preserved after repeated washing. Overall, the textile-specimen results show a trade-off between compliance and electrical continuity across the 20-cycle laundering protocol: the woven Ag/Cu/Sn fabric showed the greatest resistance stability, the silicone-coated face of Fabric 2 preserved its conductive path despite its higher absolute resistance, and the silver-plated knit and exposed knitted reverse sides showed larger electrical and visible microstructural changes.

### 3.3. Electrode Development

**Figure 7:**
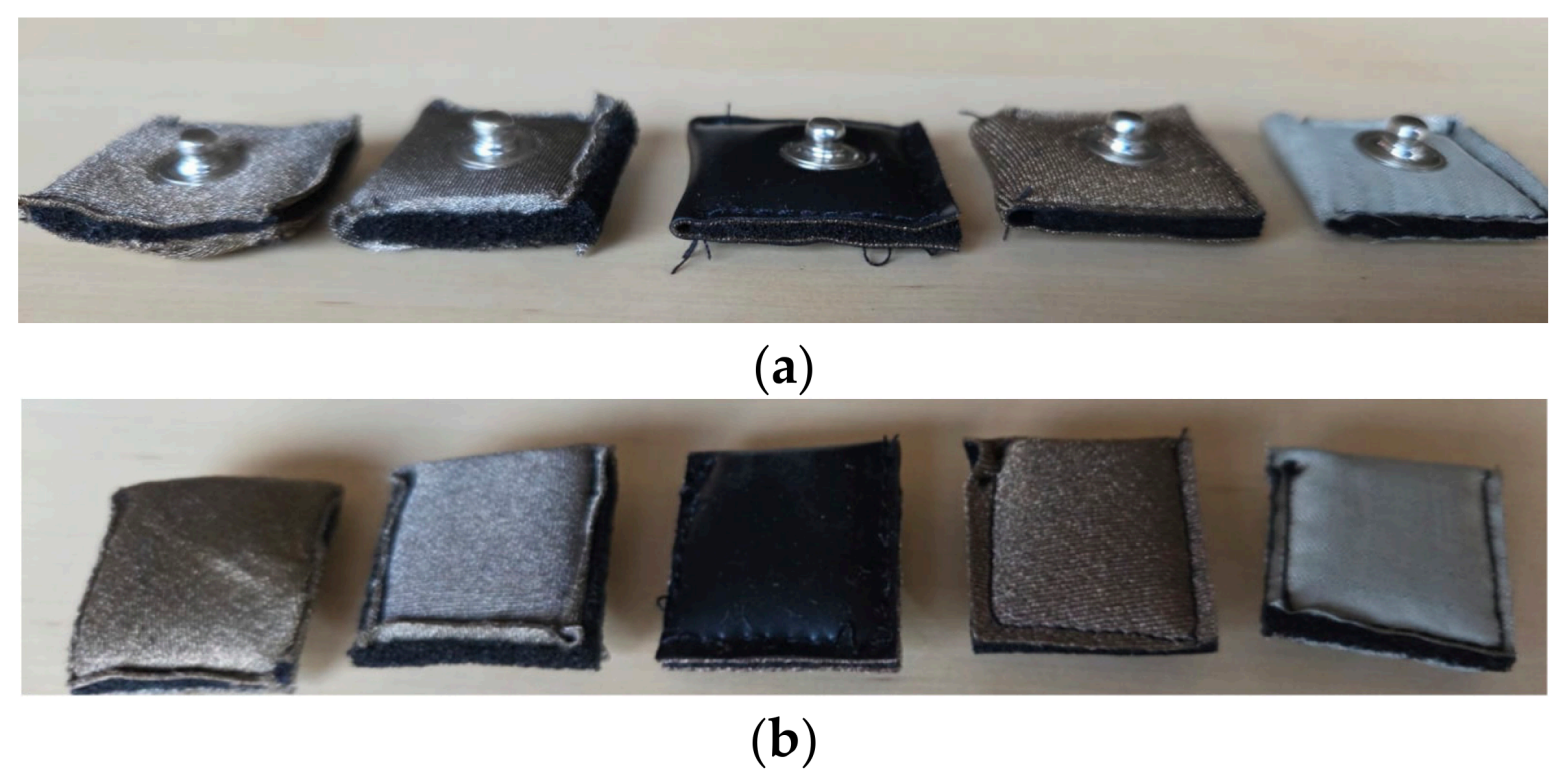
Photographs of the five textile EEG electrode prototypes derived from Fabrics 1–3. (**a**) Outer side, showing the snap connector. (**b**) Skin-contact side, showing the tested textile surface orientation and finishing.

The five electrode variants described in Section 2.4 were successfully fabricated (Figure 7). All prototypes were mechanically comparable and differed only in the conductive textile surface exposed to the skin. Washing and artificial perspiration tests were performed on corresponding constituent textile specimens with the tested surface orientations, whereas the assembled multilayer prototypes were used for electrode–phantom impedance and human EEG recordings. Complete-device washability therefore remains to be established, as no assembled prototype was laundered or immersed in artificial perspiration.

### 3.4. Electrical Characterization and Reduced Equivalent-Circuit Analysis

In the single specimens that were tested, artificial perspiration exposure produced distinct resistance responses depending on textile architecture and surface treatment (Table 5). All fabrics remained conductive under acidic, alkaline, and neutral conditions, but moisture affected each conductive pathway differently. Fabric 1 showed a pronounced resistance decrease when wet, from 0.32 ± 0.05 Ω to 0.14–0.17 Ω on the face side and from 0.35 ± 0.05 Ω to 0.17–0.20 Ω on the reverse side, consistent with electrolyte-assisted conduction and improved fiber-to-fiber contact in the silver-plated knit. Fabric 2 displayed the strongest surface asymmetry, as the silicone-coated face remained comparatively resistive despite decreasing from 40.50 ± 3.80 Ω in the dry state to 16.19–32.45 Ω after sweat exposure, while the uncoated reverse maintained much lower resistance, ranging from 1.23 ± 0.08 Ω to 1.45 ± 0.14 Ω across sweat conditions. The tested F3F specimen remained close to 0.03 Ω across all exposure media. Within this preliminary descriptive dataset, manufacturer sheet resistance alone does not capture the functional behavior of each surface: the woven Ag/Cu/Sn fabric provided the most invariant conductive path, Fabric 1 benefited from moisture-assisted low-resistance contact, and Fabric 2 showed a clear trade-off between the protective but resistive silicone-coated face and the more conductive exposed reverse.

**Figure 8:**
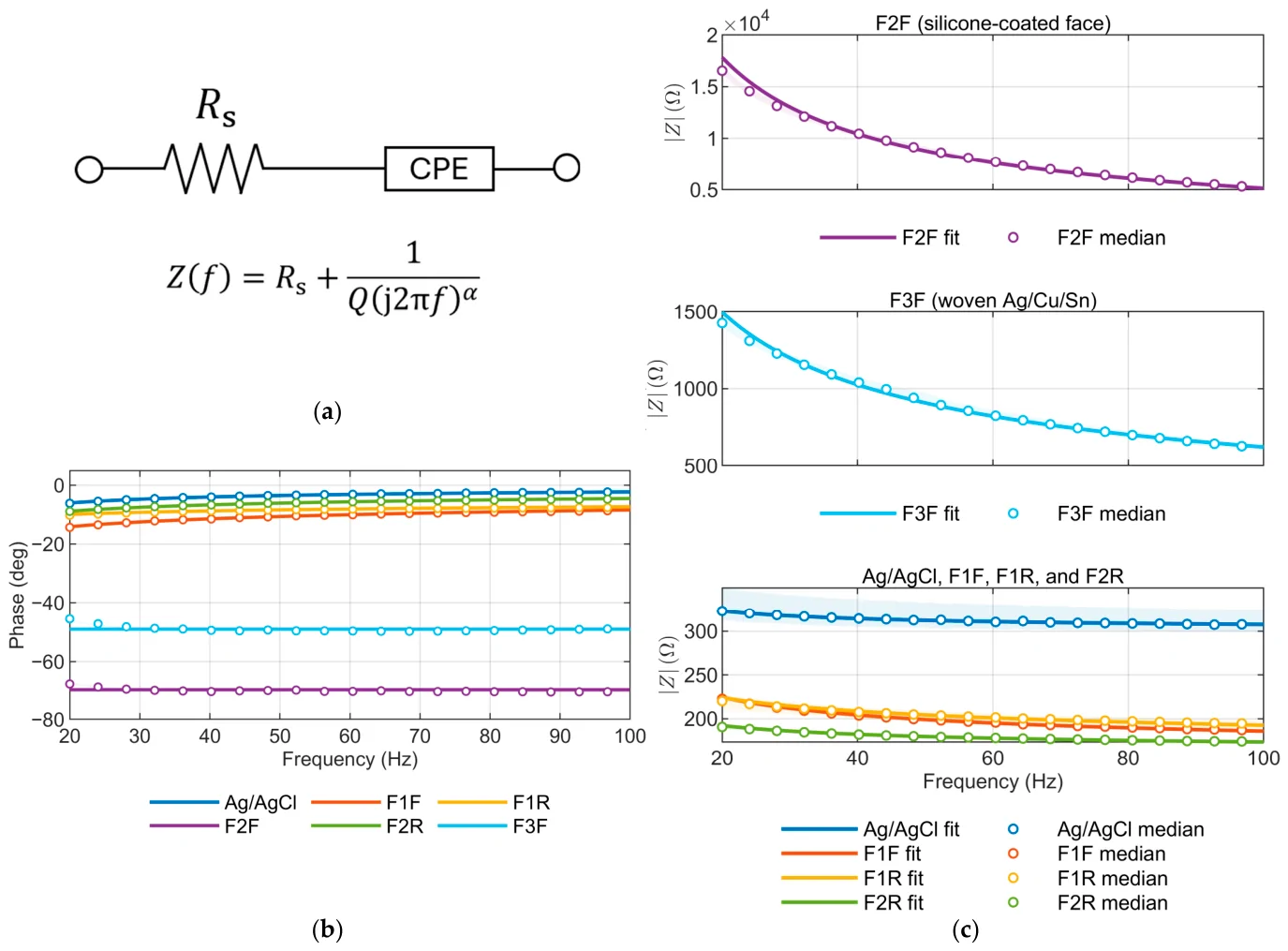
Reduced equivalent-circuit representation and electrode–phantom impedance. (**a**) Descriptive $R_{s}$–CPE model, where $R_{s}$ is an effective series contribution and the CPE admittance is *Q*(j*ω*)^α^. (**b**) Phase and (**c**) impedance magnitude from 20 to 100 Hz. Markers show the median measured complex spectrum, shaded regions show the interquartile range across complete technical sweeps obtained without changing the electrode–phantom placement, and solid lines show the reduced-model fit to the median complex spectrum.

The repeated electrode–phantom sweeps (*n* = 16 for every electrode) showed repeatable, surface-dependent differences under the fixed gelatin geometry and applied 225 g mass (Figure 8b,c). At 50 Hz, the median impedance magnitude [interquartile range] was 199 [198–201] Ω for F1F, 204 [198–209] Ω for F1R, 180 [179–180] Ω for F2R, and 313 [304–333] Ω for the Ag/AgCl comparison electrode. Their corresponding phases were −10.6° [−11.0 to −10.5°], −8.4° [−8.9 to −7.8°], −6.1° [−6.4 to −5.9°], and −3.5° [−3.6 to −3.4°]. F3F showed an intermediate magnitude of 928 [898–972] Ω with a substantially more negative phase of −49.6° [−49.8 to −49.4°], while the silicone-coated F2F surface was clearly separated from all other configurations at 8.90 [8.76–9.23] kΩ and −69.9° [−70.5 to −69.6°].

Considered together, the 50 Hz values reported above identify a pronounced orientation-dependent difference for Fabric 2, where exposing the conductive-silicone face rather than the knitted reverse increased the 50 Hz phantom impedance from 180 Ω to 8.90 kΩ, an approximately 50-fold increase, and produced a substantially more capacitive response. The face/reverse difference was much smaller for the silver-plated Fabric 1. Under the fixed phantom conditions, these measurements resolve clear architecture- and orientation-dependent differences in the controlled electrode–phantom response. Their reach is bounded in two ways: the gelatin phantom reproduces neither the stratum corneum nor the contact mechanics of an electrode held against the forehead; and the measured band begins at 20 Hz, above the delta, theta, and alpha ranges. Whether these differences translate into differences in EEG agreement is therefore addressed directly in the human recordings.

An ideal series-RC model did not reproduce the non-ideal frequency dispersion of the measured spectra: on the median spectra, its complex normalized error ranged from 1.58% to 26.84%, compared with 0.15% to 4.01% for the reduced $R_{s}$–CPE model (Table 6). The reduced model showed substantially lower BIC values than the ideal RC model for all six electrodes ($\Delta BIC_{ideal-reduced}\;$= 694.0–1103.1). Adding a parallel $R_{p}$ term did not improve BIC for five electrodes ($\Delta BIC_{full-reduced}\;$= 0.6–5.3). Only for F3F, the full model produced a lower BIC ($\Delta BIC_{full-reduced}\;$= −60.9). However, its Jacobian was singular and $R_{p}$ was poorly identified, indicating that the parameter set was not uniquely identifiable. The lower BIC was therefore not considered to be sufficient evidence to retain the more elaborate topology. The reduced model was kept as the common descriptive representation across all electrodes. The median α ranged from 0.28 to 0.77 across surfaces; the effective $R_{s}$ term was resolved for Ag/AgCl, F1F, F1R, and F2R but was not resolved within the measured band for F2F or F3F. The reduced-model parameter estimates and RC/$R_{s}$–CPE fit errors are summarized in Table 6.

### 3.5. EEG Signal Evaluation

Human EEG recordings were used to assess whether the textile electrodes preserved the main temporal and spectral characteristics of the simultaneously recorded Ag/AgCl comparison signals at Fp1. Because textile and Ag/AgCl electrodes shared the same acquisition clock, agreement was quantified on matched, sample-synchronous segments using waveform correlation, RMS amplitude agreement, blink morphology, spectral coherence, and spectral-complexity (entropy) agreement, under alternating eyes-open (EO) and eyes-closed (EC) resting conditions. Because recordings were restricted to Fp1, EO/EC alpha-band modulation was treated as a secondary descriptive check rather than as a primary validation endpoint.

**Figure 9:**
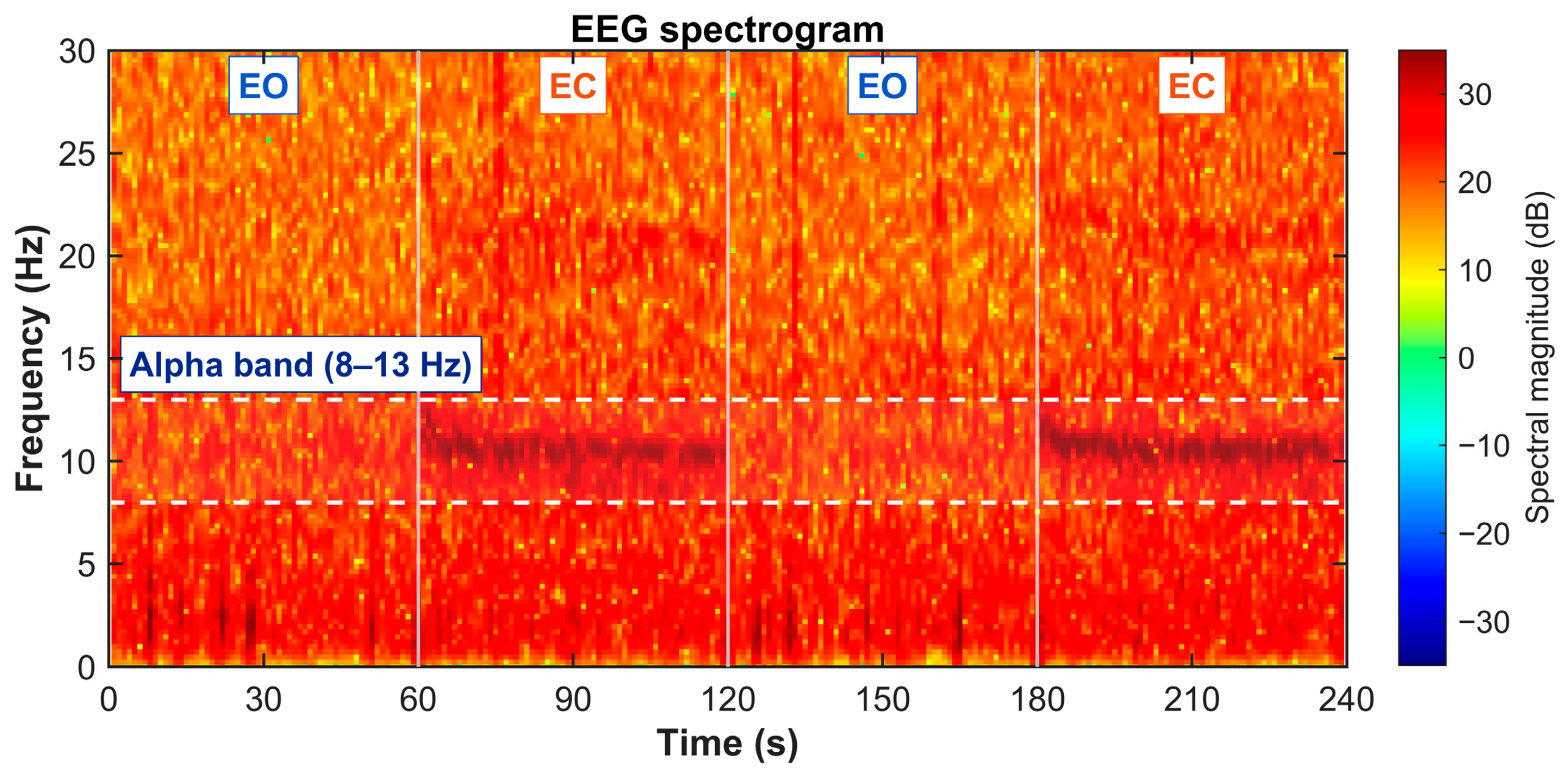
Spectrogram of EEG activity recorded with the F2R textile electrode in one representative participant. The four successive 60 s blocks are labeled EO, EC, EO, and EC, and the 8–13 Hz alpha band is indicated. The figure is illustrative and does not represent a group result.

As a qualitative check, the textile electrode captured the expected state-related change in alpha-band power: in a representative F2R recording, spectral power in the 8–13 Hz band increased during eyes-closed intervals and decreased during eyes-open intervals (Figure 9). Consistent with the frontal recording site, this EO/EC contrast was present but variable across subjects, in line with the predominantly posterior origin of the alpha rhythm; it is therefore reported as a descriptive observation rather than as a quantitative endpoint.

A separate feasibility pilot (n=4), conducted before the full campaign using the same OpenBCI Cyton acquisition system, included the silicone-coated F2F surface. Across all four participants, F2F showed lower agreement with the simultaneously recorded Ag/AgCl comparison signals than the other candidate surfaces examined in the pilot, with temporal correlations of 0.58 ± 0.30 during EO and 0.50 ± 0.28 during EC, as well as spectral coherence of 0.52 ± 0.22 during EO and 0.47 ± 0.27 during EC. These observations coincided with the high and strongly capacitive F2F phantom response. However, no prespecified numerical carry-forward criterion was used, and F2F was not evaluated in the subsequent n=9 cohort. The decision not to carry F2F forward was therefore outcome-informed. The pilot is reported as exploratory, is not pooled with the full-cohort estimates, and does not establish that the phantom response caused the poorer EEG agreement. The main analysis is explicitly limited to F1F, F1R, F2R, and F3F.

This outcome contrasts with a concurrent ECG study, in which the same conductive-silicone stretch-knit fabric showed the best aggregate performance against an Ag/AgCl reference, although no significant between-material differences were detected [36]. The contrast may reflect modality- and protocol-specific differences in placement, geometry, fixation, instrumentation, and analysis, but the present data do not isolate the mechanism. The higher-impedance, more capacitive F2F phantom response cannot be transferred directly to the in vivo interface or treated as causal. Electrode suitability should therefore be validated in the intended recording configuration rather than inferred across modalities; in this study, the lower-impedance uncoated reverse (F2R) was retained for EEG.

**Figure 10:**
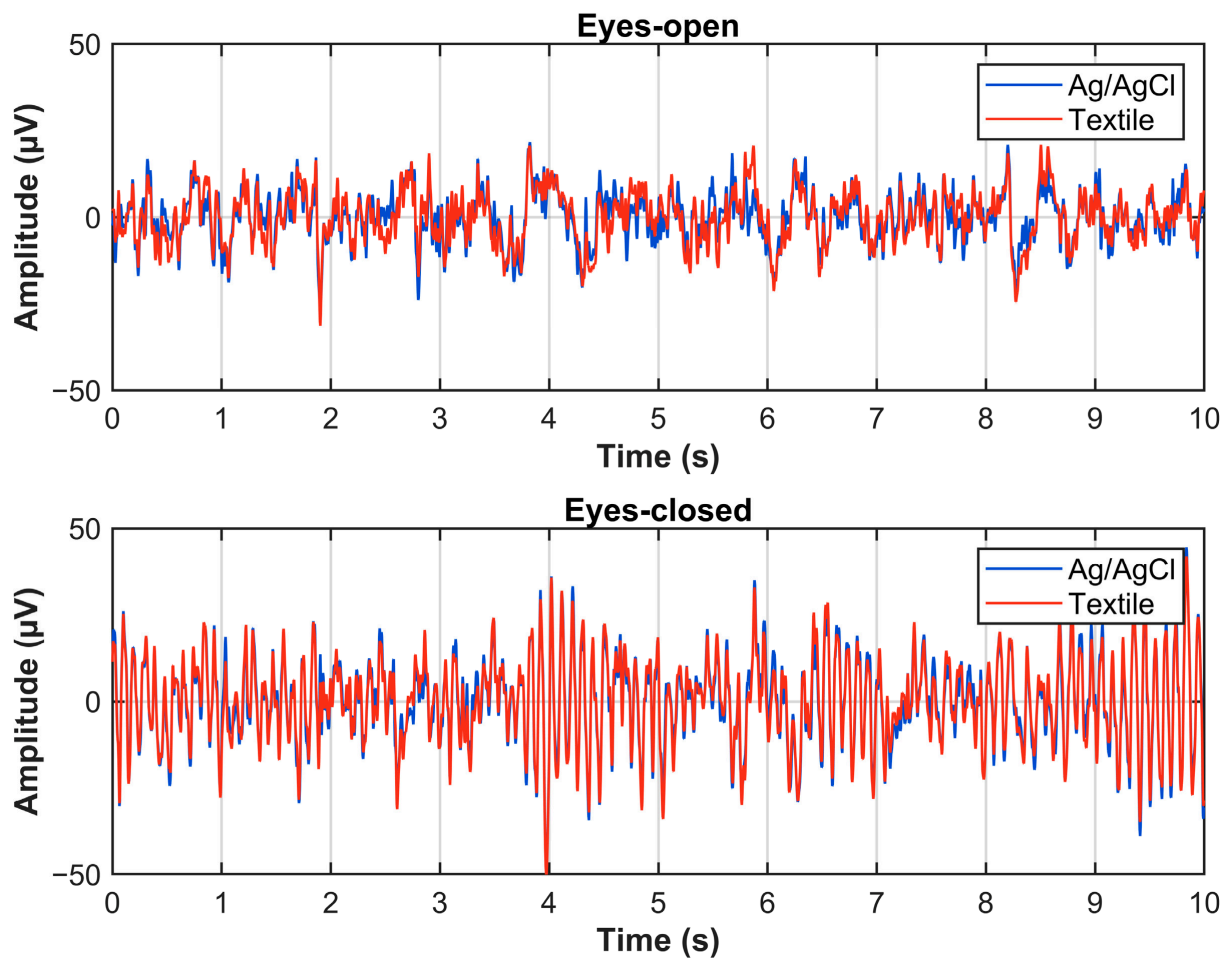
Representative simultaneous EEG recording from one participant and one F1R session during eyes-open and eyes-closed conditions. Preprocessed waveforms recorded with a commercial Ag/AgCl comparison electrode (blue) and textile electrode F1R (red) from a representative subject during eyes-open (upper panel) and eyes-closed (lower panel) conditions, each spanning 10 s.

Representative simultaneous traces from one participant and one F1R session showed close temporal overlap between the textile and Ag/AgCl comparison signals in both eyes-open and eyes-closed conditions (Figure 10): lower-amplitude oscillations during eyes-open (~20–40 µV peak-to-peak) and higher-amplitude, more regular oscillations during eyes-closed (~40–80 µV peak-to-peak), supporting the subsequent quantitative waveform-agreement analysis.

**Figure 11:**
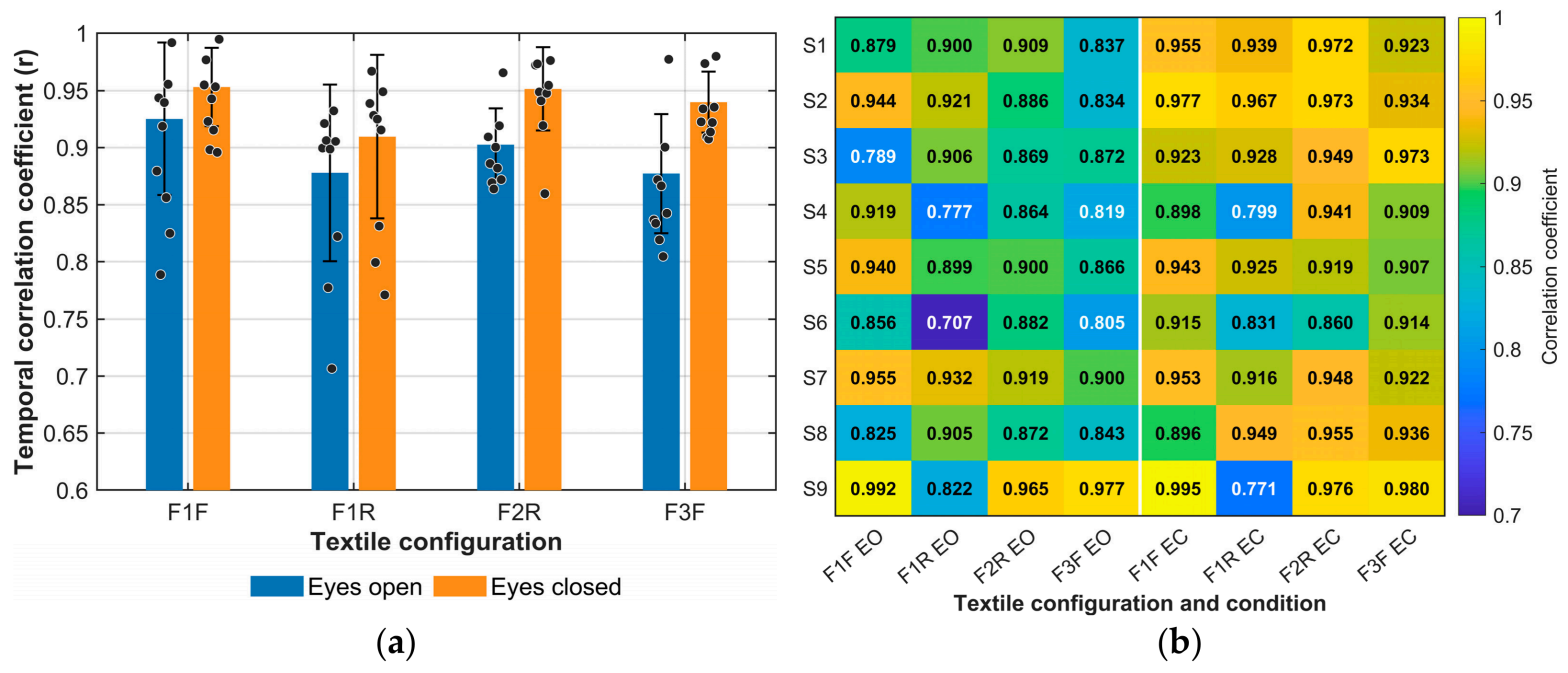
Temporal correlation between the textile and Ag/AgCl comparison signals. (**a**) Back-transformed mean correlation (±standard deviation across *n* = 9 subjects) for each textile configuration during eyes-open (EO) and eyes-closed (EC) conditions; overlaid points show individual subject values. (**b**) Individual subject correlation coefficients across all configurations and conditions, shown as a heatmap (*n* = 9). No configuration effect was detected within this sample in the primary mixed model.

Group mean correlations ranged from *r* = 0.877 to *r* = 0.953 across textile configurations and conditions (Table 7, Figure 11a). These high correlations indicate that the textile electrodes closely reproduced the temporal waveforms of the Ag/AgCl comparison signals. Temporal correlation reflects waveform similarity rather than quantitative interchangeability; amplitude and spectral agreement are therefore assessed separately through the Bland–Altman, concordance, and equivalence analyses below.

In the primary mixed model on Fisher-*z* correlations, no configuration effect was detected within this sample, F(3, 63) = 1.84, *p* = 0.149, nor was the condition × configuration interaction, F(3, 63) = 0.63, *p* = 0.596. This absence of a detected effect is not evidence that the four textile surfaces perform equivalently. The main condition effect was sensitive to the assumed covariance structure—non-significant in the mixed model (F(1, 63) = 3.51, *p* = 0.066) but significant in a repeated-measures ANOVA sensitivity analysis (F(1, 8) = 20.90, *p* = 0.002)—because the condition is not central to electrode comparison, this discrepancy does not alter the conclusion of high temporal agreement across configurations.

Mean correlations were uniformly high across configurations, and every configuration reached its highest values during eyes-closed. All 72 individual subject–configuration–condition correlations exceeded *r* = 0.70, with only a small number below *r* = 0.80 (Figure 11b), indicating consistently high waveform fidelity across participants and surfaces. Because configuration was not a significant predictor, these descriptive differences are not interpreted as establishing the superiority of any single configuration.

The grand-mean temporal correlation across subjects was *r* = 0.916 (between-subject SD = 0.033; range of subject means 0.857–0.969), indicating that the textile electrodes closely reproduced the temporal dynamics of the Ag/AgCl comparison signals. This level of agreement is consistent with published validation studies of textile and dry EEG electrodes, which report temporal correlations against wet references frequently exceeding *r* = 0.80 for frontal placements [6,7,12,13].

**Figure 12:**
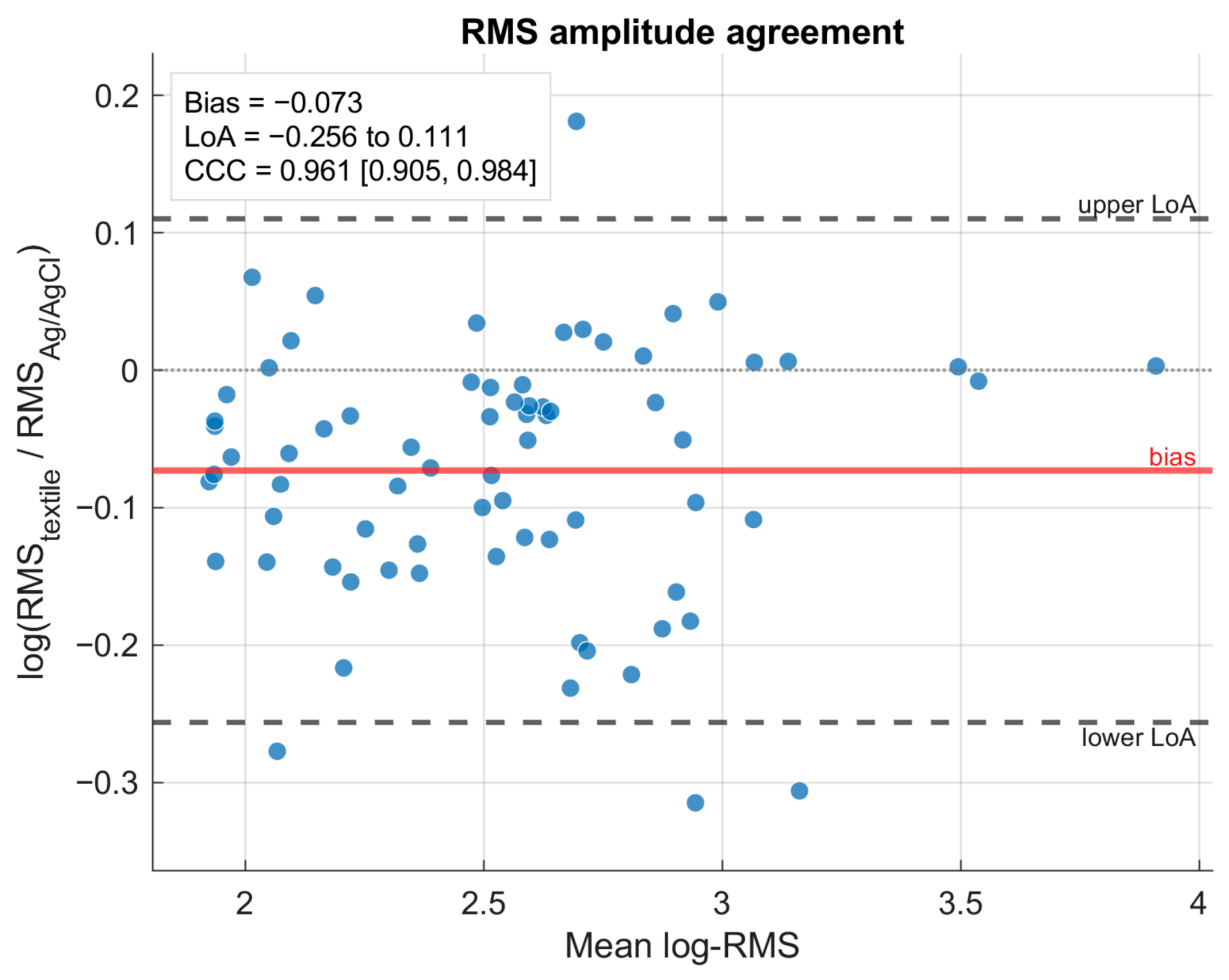
Repeated-measures Bland–Altman plot for RMS amplitude agreement between the textile signals and the Ag/AgCl comparison signals. Each point is one subject–configuration–condition observation; the *x*-axis is the mean log-RMS of the two electrodes and the *y*-axis their difference on the log scale, log(RMS_textile_/RMS_Ag/AgCl_). The solid line marks the global bias (−0.073 log units, −7.0%) and the dashed lines mark the repeated-measures 95% limits of agreement (−0.256 to 0.111 log units). Negative values indicate lower amplitude in the textile recording. No significant proportional bias was detected (slope = −0.038, *p* = 0.221), so the limits are constant across the amplitude range.

RMS amplitude agreement was assessed within a method-comparison framework (RMS log-ratio, repeated-measures Bland–Altman, CCC, and TOST; see Section 2.7). Across all subjects and configurations, textile RMS was modestly and systematically lower than that of the Ag/AgCl comparison signals, with a global log-ratio bias of −0.073 (−7.0%) and repeated-measures 95% limits of agreement from −0.256 to 0.111 log units (−22.6% to +11.7%; Figure 12). Concordance was high (CCC = 0.961, 95% bootstrap CI 0.905–0.984). Exploratory TOST supported equivalence of the average bias at ±15% and ±20% margins (*p* = 0.002 and *p* < 0.001) but not at ±10% (*p* = 0.084), consistent with a small systematic attenuation.

No configuration effect on the RMS log-ratio was detected within this sample: per-configuration mean differences ranged from −5.0% to −9.6%, were directionally consistent across conditions, and showed no amplitude inflation in any configuration.

The −7% bias was small compared with the spread of the individual differences (95% limits of agreement −22.6% to +11.7%), and the waveforms themselves were preserved, with temporal correlations remaining high across all configurations. However, it must be interpreted as a system-level difference between the complete simultaneous recording configurations. The textile and Ag/AgCl sensing surfaces were adjacent rather than co-located and used different attachment principles, and contact force was not instrumented or matched. The attenuation, therefore, cannot be assigned uniquely to intrinsic signal loss in the conductive fabric. Within subjects, RMS attenuation was not associated with temporal-shape fidelity ($r_{rm}$ = −0.161, 95% CI −0.392 to 0.088, *p* = 0.202), consistent with amplitude agreement and waveform correlation describing complementary properties rather than identifying the physical origin of the bias.

Blink morphology provided an additional check of transient waveform capture at Fp1. Blinks were detected during eyes-open blocks at a 100 µV amplitude threshold (with 150 and 200 µV as sensitivity checks; see Section 2.7), and per-event textile–Ag/AgCl correlations were averaged on the Fisher-*z* scale so that each subject contributed a single value. Across the nine subjects, back-transformed blink correlations were uniformly high (0.993 ± 0.004 for F1F, 0.991 ± 0.006 for F1R, 0.990 ± 0.006 for F2R, 0.992 ± 0.004 for F3F), indicating close preservation of blink morphology by all textile surfaces. Textile-to-Ag/AgCl peak-amplitude ratios ranged from 0.82 to 0.90 and per-blink RMSE from 16 to 23 µV, consistent with the small amplitude attenuation observed for RMS. No configuration effect on blink-correlation fidelity was detected at 100 µV (Friedman χ^2^(3) = 1.8, *p* = 0.61), and this conclusion was unchanged at the 150 µV (χ^2^(3) = 4.65, *p* = 0.20, *n* = 8) and 200 µV (χ^2^(3) = 1.56, *p* = 0.67, *n* = 5) thresholds.

**Figure 13:**
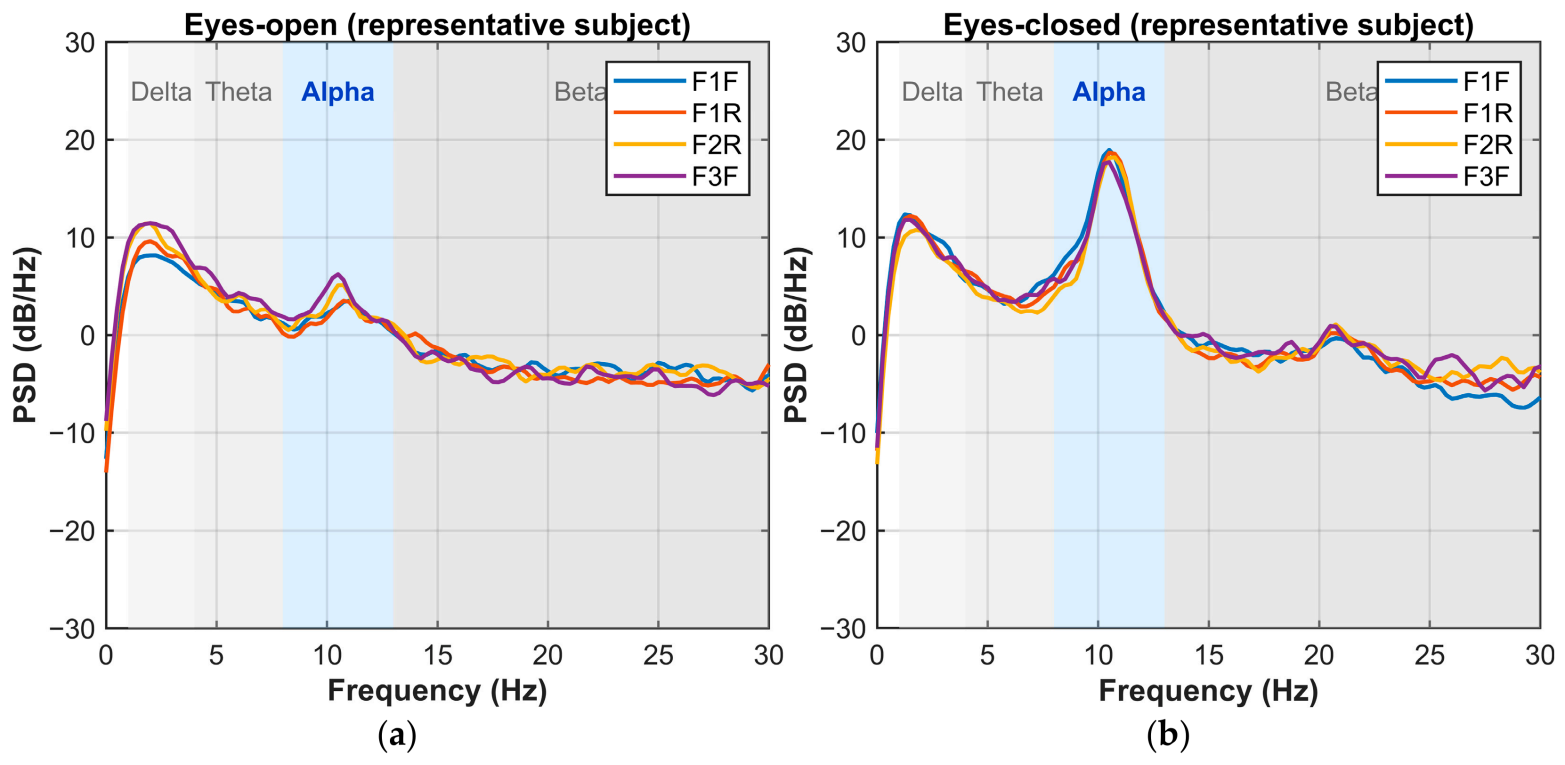
Power spectral density from one representative participant. Condition-specific Welch PSD estimates for F1F, F1R, F2R, and F3F are shown during (**a**) eyes-open and (**b**) eyes-closed conditions. The curves are illustrative and do not represent group averages.

Power spectral density in a representative recording showed the characteristic low-frequency-dominant EEG profile for all textile configurations, with a clear increase in the alpha band (8–13 Hz) during eyes-closed relative to eyes-open (Figure 13). The Welch PSD estimates from F1F, F1R, F2R, and F3F overlapped closely across the spectrum in both conditions, indicating consistent spectral estimates across configurations. As noted above, this eyes-closed alpha increase was clear in this representative subject but variable across the cohort, consistent with the predominantly posterior origin of the alpha rhythm. Quantitative spectral agreement with the Ag/AgCl comparison signal across subjects was then evaluated by coherence.

As a secondary check, the eyes-closed/eyes-open alpha power ratio was compared between electrode types. Mean alpha reactivity was similar for Ag/AgCl (4.71 ± 4.32) and textile (4.81 ± 4.48) recordings, with no significant difference (paired analysis *p* = 0.593), and both electrode types showed consistent paired EO/EC behavior, including one participant with an inverted ratio captured by both systems. Because the recording was restricted to Fp1, where alpha reactivity is inherently modest, this measure is reported as a descriptive consistency check rather than as a quantitative validation endpoint.

Spectral agreement was evaluated as band-averaged magnitude-squared coherence between textile and Ag/AgCl comparison signals across delta, theta, alpha, and beta bands in *n* = 9 participants (288 coherence estimates; analyzed on the logit scale, see Section 2.7). Averaged across bands, configurations, and conditions, the mean coherence was 0.86 on the original [0, 1] scale, with pronounced differences between frequency bands.

In the primary mixed model on logit-transformed coherence (Table 8), the band was a strong predictor, *F*(3, 279) = 12.0, *p* = 2.1 × 10^−7^, whereas no configuration effect was detected within this sample, *F*(3, 279) = 1.57, *p* = 0.196. This absence of a detected effect is not evidence that spectral agreement was equivalent across the four textile surfaces. The nominal condition effect did not survive family-wise adjustment (*p* = 0.025 raw; *p* = 0.151 adjusted) and was interpreted only as a weak tendency toward higher coherence with eyes closed. No interaction term was significant, including the three-way configuration × condition × band interaction (*p* = 0.999); in particular, the absence of a band × configuration interaction indicates that no evidence was found that any configuration performed selectively better or worse in a specific band.

**Figure 14:**
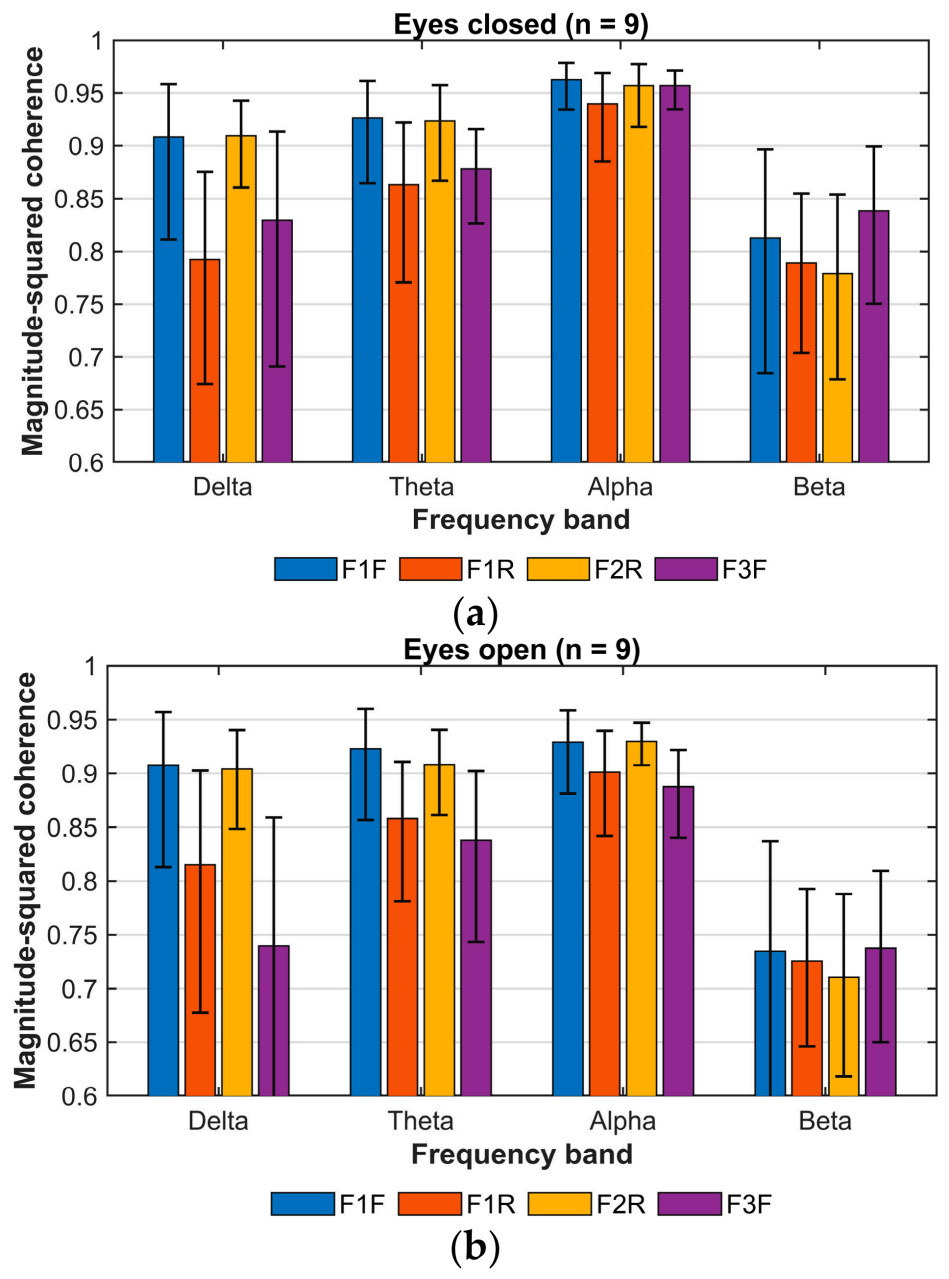
Spectral coherence between the textile and Ag/AgCl comparison signals across four frequency bands (delta, theta, alpha, and beta) and textile configurations during eyes-closed (**a**) and eyes-open (**b**) conditions (*n* = 9). Bars show mean magnitude-squared coherence while error bars indicate the standard error of mean. The band was a significant predictor of coherence, whereas no configuration effect was detected within this sample.

Descriptively (original coherence scale), mean coherence was highest in alpha (0.93), followed by theta (0.89), delta (0.85), and beta (0.77) (Figure 14). Alpha-band coherence exceeded 0.80 for all configurations and conditions, whereas delta coherence, although high on average, fell below 0.80 in isolated cells, and beta was consistently the lowest band. These results indicate high spectral agreement in the lower EEG bands and the alpha range, decreasing toward beta frequencies, consistent with previous reports on dry and textile EEG electrodes describing reduced signal-to-noise ratio and greater artifact susceptibility at higher frequencies [4,5,6,7].

Spectral entropy showed similar group means for Ag/AgCl (0.812 ± 0.085) and textile (0.803 ± 0.082) recordings, but agreement was the least precise of the metrics examined. The global repeated-measures bias was −0.087 on the logit scale, with 95% limits of agreement from −0.613 to 0.438, and Lin’s CCC was 0.891 (95% bootstrap CI 0.724–0.962)—the lowest concordance and widest limits of agreement of any metric.

**Figure 15:**
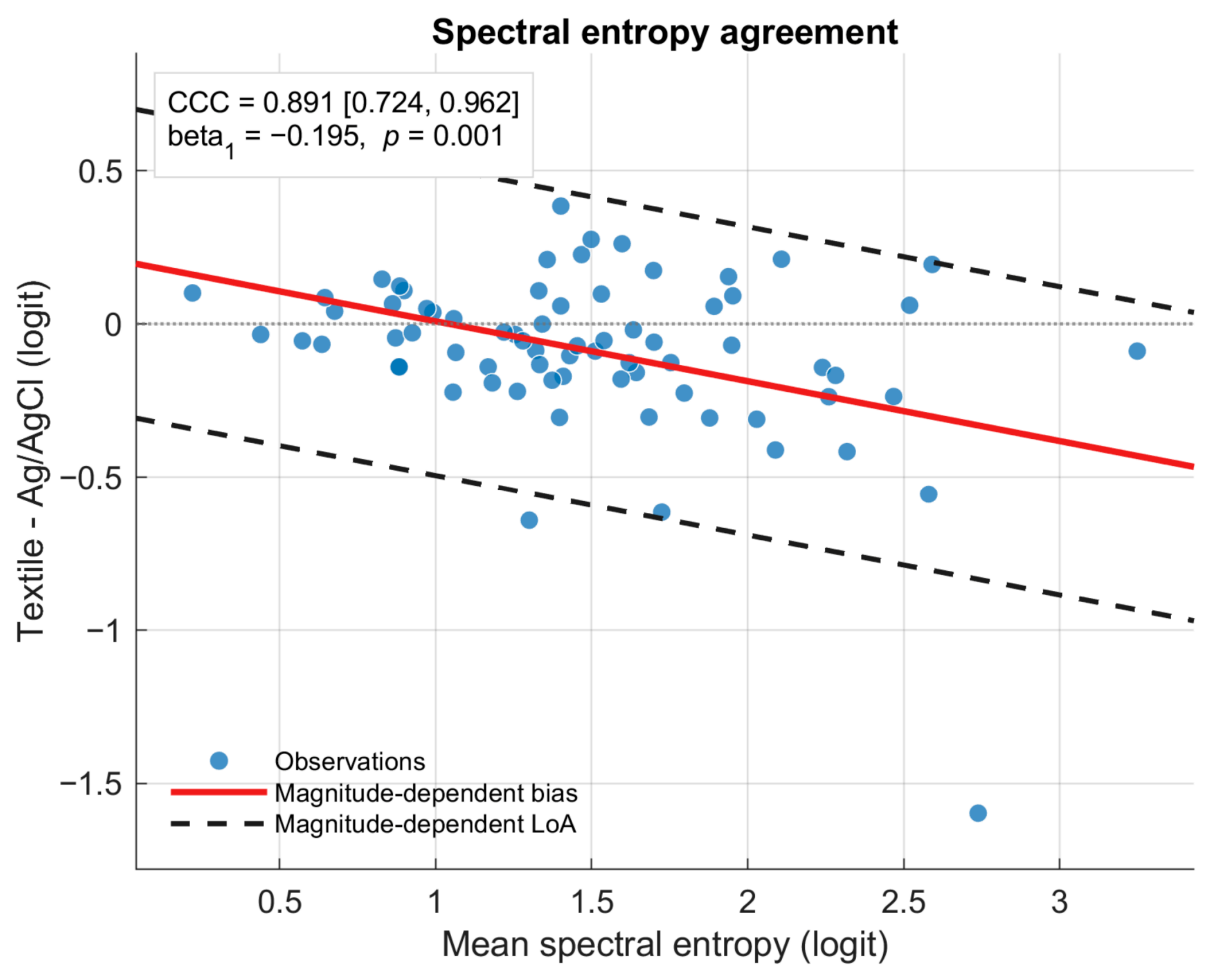
Repeated-measures Bland–Altman plot for spectral entropy agreement between the textile and Ag/AgCl comparison signals on the logit scale. Each point is one subject–configuration–condition observation; the *x*-axis is the mean entropy of the pair and the *y*-axis is their difference (textile minus Ag/AgCl comparison signal). Because entropy showed a significant proportional bias (slope = −0.195, *p* = 0.001), the bias line (solid) and 95% limits of agreement (dashed) vary with entropy magnitude rather than being constant. Entropy was the metric with the lowest concordance (CCC = 0.891).

Entropy also showed a significant proportional bias—the only metric for which this occurred—with the textile–Ag/AgCl difference varying systematically with mean entropy (magnitude-dependent slope β_1_ = −0.195, *p* = 0.001; Figure 15). The bias shifted from slightly positive at low entropy to progressively negative at high entropy (from +0.038 to −0.243 logit across the 10th–90th percentiles), so the limits of agreement depend on entropy magnitude rather than being constant, and entropy differences should be interpreted relative to the underlying entropy level.

Across configurations, the reverse weft-knit side F1R showed the weakest concordance (CCC = 0.787, 95% CI 0.474–0.942) while the others were higher (CCC 0.908–0.957); however, no configuration effect on the paired logit difference was detected after multiplicity correction (*F*(3, 72) = 2.18, *p* = 0.293 adjusted), so this variation is regarded as exploratory. Exploratory TOST on the raw normalized-entropy scale supported equivalence of the average bias at ±0.02 and ±0.05 margins but not at ±0.01. Overall, entropy was broadly preserved but was the least precisely reproduced metric, and entropy-based measures from textile recordings should be interpreted with this magnitude-dependent agreement in mind.

Taken together, the retained textile electrodes showed high agreement with the simultaneously recorded Ag/AgCl comparison signals at Fp1 across the time-domain and spectral measures. Agreement was strongest for temporal waveform correlation (grand-mean *r* = 0.916) and blink morphology, high for RMS amplitude (CCC = 0.961, with a small −7% attenuation), and high albeit frequency-dependent for spectral coherence, which decreased from the alpha and lower bands toward beta. Entropy was broadly preserved but the least precisely reproduced, with the lowest concordance and a magnitude-dependent bias. Within the limits of this preliminary Fp1 dataset, no configuration effect was detected for the main EEG agreement metrics, and no retained configuration showed consistent superiority; neither result is interpreted as evidence of equivalent performance. Across the impedance range spanned by the retained surfaces, the phantom characterizations of Section 3.4 did not rank the surfaces in the order of their EEG agreement: F3F combined an intermediate but strongly dispersive phantom response with agreement comparable to the other retained surfaces. Material selection should therefore also consider secondary criteria, such as textile-level laundering durability, comfort, and manufacturability.

### 3.6. Limitations

This study has limitations related to the study cohort and recording site. The sample was small (*n* = 9 healthy young adults), and recordings were restricted to a single frontal site (Fp1) under resting conditions; alpha reactivity at the frontal sites was inherently modest given the predominantly posterior origin of the alpha rhythm, so the EO/EC alpha contrast was treated only as a descriptive consistency check. Moreover, Fp1 is a hairless, easily accessible site, which represents a comparatively favorable scenario for dry textile contact; therefore, the present results do not yet establish performance over hair-bearing scalp regions, where dry electrodes must overcome the additional barrier of hair and where textile surfaces without protruding structures may couple less effectively. Validation at hair-covered sites, in larger and more diverse cohorts, is an important step still pending. F2F was not evaluated in the same *n* = 9 cohort as the other four configurations; that decision was informed by an exploratory four-participant pilot without a prespecified numerical criterion, so the study does not provide a confirmatory same-cohort comparison of all five prototypes.

Further limitations concern the measurement conditions. The dry textile and pre-gelled Ag/AgCl electrodes were secured by their respective attachment principles—an elastic headband and an integrated adhesive—and the two sensing surfaces were adjacent rather than co-located, so contact force was neither instrumented nor matched between electrode types. The −7% RMS bias therefore represents a difference between the complete recording configurations rather than an intrinsic textile attenuation coefficient, and instrumented contact force would allow a more rigorous like-for-like comparison. While the order of the four textile configurations was randomized across participants, with nine participants, the exact balance of every configuration across all four period positions was not possible, so residual period or carry-over effects—including fatigue, habituation, progressive hydration, and local interface changes caused by replacing the textile prototype—cannot be excluded. All recordings were acquired at rest during approximately 20 min sessions, without motion tasks, repeated donning, long-duration wear, or a validated comfort and usability assessment.

The aging experiments also had a restricted scope. Laundering was performed on *n* = 3 constituent textile specimens per fabric rather than on complete multilayer electrodes, so foam integrity, seams, connector fixation, and interlayer electrical continuity were not evaluated, and complete-device washability was not demonstrated. Laundering followed a single standardized cycle (procedure 9B, cold water with normal agitation, 20 repetitions); warmer domestic cycles were not evaluated. The artificial perspiration measurements used one independent specimen per fabric–surface–condition combination, with the nine readings acquired as technical repetitions from that specimen; these data are therefore preliminary and descriptive and do not estimate specimen-to-specimen variability. Resistance and SEM characterize electrical continuity and visible morphology but do not identify oxidation potential, corrosion kinetics, charge-transfer resistance, or other electrochemical degradation mechanisms.

The impedance characterization was constrained in both frequency range and test medium. The B&K Precision 895 LCR meter imposed the 20 Hz lower measurement limit, whereas 100 Hz was the upper limit selected in the original EEG-oriented protocol; the resulting spectra therefore do not cover the delta, theta, and alpha ranges in which coherence agreement was highest, and no extrapolation below the measured interval was performed. The two-electrode gelatin phantom provides a standardized comparison of electrode–phantom behavior but does not reproduce the stratum corneum, hair, local perspiration, non-uniform pressure, or micro-motion, so the absolute values reported here should not be read as in vivo scalp impedance. Because the phantom impedance was used only for a controlled between-electrode comparison rather than as an absolute in vivo estimate, this range restriction does not affect the EEG agreement results, which were derived directly from the human recordings across the full analysis band (~1–45 Hz).

## 4. Conclusions

This work shows that commercially available metallized textiles, evaluated with standardized textile and electrophysiological protocols, are promising dry EEG electrode candidates that do not require custom laboratory fabrication. Fabric architectures and face/reverse surface orientations emerged as practical design parameters influencing textile-level laundering durability, resistance behavior, and the controlled electrode–phantom response, while the retained textile configurations showed high agreement with the simultaneously recorded Ag/AgCl comparison signals in this preliminary Fp1 dataset.

The three textile constructions showed distinct durability profiles. The woven PA66 fabric with Ag/Cu/Sn metallization exhibited the greatest resistance stability across 20 laundering cycles, whereas the silver-plated knit and exposed knitted surfaces were more affected by repeated laundering. These results emphasize a trade-off between conformability and long-term electrical stability, which should be considered when selecting textile materials for washable EEG electrodes; they apply to constituent textile specimens and do not demonstrate the washability of the complete multilayer electrodes. Surface orientation also had a pronounced effect on the controlled electrode–phantom response, the conductive-silicone F2F surface showing substantially higher impedance magnitude and a more capacitive phase response than the F2R reverse, although impedance alone did not rank the retained surfaces in the order of their EEG agreement.

Across time-domain and spectral agreement metrics, the retained textile surfaces closely matched the comparison signal, with high temporal waveform agreement, a small systematic RMS amplitude attenuation, and frequency-dependent spectral coherence that was highest in the lower-frequency and alpha bands. The adjacent sensor positions, distinct fixation methods, and unmeasured contact force prevent this bias from being attributed uniquely to the textile material. Entropy was broadly preserved but showed wider limits of agreement and magnitude-dependent bias, indicating that spectral-complexity measures derived from textile recordings should be interpreted with appropriate caution. Together, these findings support a textile-centered and reproducible pathway for selecting and qualifying conductive fabrics for scalable, washable, and wearable EEG systems.

Among the four retained configurations (F1F, F1R, F2R, and F3F), no configuration effect on the main EEG agreement metrics was detected in the primary models. Within the limits of this preliminary dataset, no retained textile surface showed consistent superiority across the main EEG agreement outcomes, and neither result was interpreted as evidence of equivalent performance. Therefore, material selection should consider EEG agreement together with practical criteria such as washability, comfort, manufacturability, and long-term mechanical stability.

This first qualification stage establishes the textile-level durability, controlled-interface, and human-agreement baseline on which the remaining validation can be built. Three lines of work follow directly from it: completing the durability chain, by laundering complete assembled electrodes and characterizing coating aging with independent specimens and broad-band electrochemical methods; instrumenting the interface, through matched contact force and in vivo skin–electrode impedance measurements; and extending the recording conditions, through motion and ambulatory tasks, longer wearing periods with standardized comfort and usability assessment, and multisite montages, with hair-bearing sites requiring a hair-traversing contact architecture. These are the main steps required to translate commercial metallized textiles into practical wearable EEG systems.

## Figures and Tables

**Table 1:** Comparison of the principal wet, semi-dry, and dry electrode–scalp coupling strategies relevant to EEG. Categories are practical rather than mutually exclusive because electrode material and contact geometry may be combined in a single device. Characteristics are representative and depend on materials, geometries, fixation, front-end electronics, and experimental protocols.

Electrode Strategy	Coupling and Typical Application	Main Advantages	Main Limitations
Conventional wet EEG electrodes [2,6,7,27]	Ionic coupling through conductive gel, paste, or hydrogel; hairless skin or prepared scalp	Established EEG technology and common comparator in validation studies; generally stable coupling; mature protocols	Skin preparation and cleaning; electrolyte application; drying or spreading; irritation; consumables and longer setup time
Semi-dry [9,27]	Limited electrolyte delivered by an absorbent element, porous probe, or internal reservoir; hairless or hair-bearing use depending on geometry	Less electrolyte and faster preparation than conventional wet electrodes; generally lower contact impedance than fully dry contact	Electrolyte depletion, leakage, or uncontrolled release; maintenance; finite operating duration; pressure-dependent delivery
Planar direct-contact dry, including textiles, conductive polymers, and foams [11,12,13,14,26,27,28]	Direct contact maintained by surface conformity, contact area, fixation pressure, and natural skin moisture; mainly hairless or low-hair sites	Gel-free and potentially reusable; soft and conformable variants can be integrated into garments or headbands; scalable fabrication	Sensitive to pressure, hydration, movement, local geometry, and hair barrier; durability depends strongly on coating and textile structure
Structured direct-contact dry, including pins, combs, and textile hooks [27,29,30,31]	Protruding structures traverse hair and establish localized scalp contact without intentionally penetrating the skin	Dry acquisition at hair-bearing sites; reduced need for scalp preparation	Force- and geometry-dependent performance; possible discomfort; sensitivity to cap fit, movement, and cleaning
Microneedle or minimally invasive dry [32]	Microprojections penetrate or bypass the high-impedance stratum corneum; applicability depends on array geometry	Very low interface impedance; reduced influence of the outer skin barrier	Minimally invasive contact; sterilization, mechanical breakage, biocompatibility, user-acceptance, and regulatory requirements
Capacitive or non-contact [5,27,33]	Dielectric coupling through an insulating layer, clothing, hair, or an air gap; requires a very-high-input-impedance front end	No electrolyte or galvanic skin contact; minimal skin preparation	Very high source of impedance; strong sensitivity to motion and environmental interference; demands shielding and front-end electronics

**Table 2:** Manufacturer-declared nominal specifications of the conductive textiles used for EEG electrode fabrication [37,38,39]. Values in parentheses are the manufacturer’s tolerances and are not experimental standard deviations. “Not specified” indicates that the corresponding value was absent from the manufacturer’s data sheet. In the “weave/knit type” column, the commercial fabric designation is followed by the structural classification, which was determined in this work by stereomicroscopic examination of the face and reverse of each fabric (Section 3.1).

Fabric	Commercial Name	Polymer Composition	Weave/Knit Type	Metal Coating	Manufacturer-Declared Composition	Areal Density (g/m^2^)	Thickness (mm)	Surface Resistivity (Ω/sq)	Temperature Range (°C)
Fabric 1	Shieldex^®^ Med-tex P180	94% Polyamide (PA) + 6% Elastane (EL)	Mirror-satin, weft-knit	Silver	73% PA/4% EL/23% Ag (±10%)	205 (±20%)	0.55 (±15%)	Not specified	−30 to 80
Fabric 2	Shieldex^®^ Silitex	78% Polyamide (PA) + 22% Elastomer	Stretch-tricot, warp-knit	Silver + conductive silicone	26.9% Ag (±1%)	380 (±10%)	0.65 (±10%)	<5	−30 to 90
Fabric 3	Shieldex^®^ Zell RS	100% Polyamide/Nylon 6.6 (PA66)	Ripstop, woven	Silver + copper + tin	10.39% Ag/40.16% Cu/4% Sn	88 (±15%)	0.11 (±15%)	<0.02	−30 to 90

**Table 3:** Temporal and spectral parameters used for EEG signal evaluation and the corresponding statistical tests.

Parameter	Definition (Per Subject)	Range/Units	Transformation for Inference	Primary Test
Temporal correlation	Pearson *r* between textile and Ag/AgCl comparison signals (artifact-free)	*r* ∈ [−1, 1]	Fisher-*z*, atanh(*r*)	Mixed model: *z* ~ Condition × Configuration + (1|Subject); RM-ANOVA as sensitivity
RMS amplitude	Paired RMS agreement, log(RMS_textile_/RMS_Ag/AgCl_); also reported as ΔRMS (%)	log-ratio; reported as % via 100(exp(*D*) − 1)	Already analyzed as log-ratio	Mixed model: *D* ~ Condition × Configuration + (1|Subject); repeated-measures Bland–Altman, CCC, and exploratory TOST ±10/15/20%
Blink morphology	Per-blink Pearson *r* in ±500 ms windows; summarized per subject × configuration as mean Fisher-*z*	*r* ∈ [−1, 1]; Fisher-*z* unbounded	Mean of atanh(*r_i_*); back-transform for descriptive *r*	Friedman on subject-level Fisher-*z* mean at 100 µV; mixed model as sensitivity; 150/200 µV as threshold sensitivity
Spectral coherence	Band-averaged magnitude-squared coherence between textile and Ag/AgCl comparison signals; delta, theta, alpha, beta	*c* ∈ [0, 1]	logit(*c*), clipped	Mixed model: logit(*c*) ~ Condition × Band × Configuration + (1|Subject); RM-ANOVA with GG as sensitivity
Spectral entropy	Normalized Shannon entropy of PSD (1.0–45 Hz)	SE ∈ [0, 1]	logit(SE), clipped	Mixed model on logit difference; repeated-measures Bland–Altman, with magnitude-dependent LoA if proportional bias; CCC; and exploratory raw-scale TOST ±0.01/0.02/0.05
Alpha reactivity	Eyes-closed/eyes-open alpha-power ratio (8–13 Hz); compared between electrode types	ratio	log-ratio	Secondary descriptive comparison (paired test of EC/EO ratio)

**Table 4:** Electrical resistance (Ω) of conductive fabrics measured before washing and after 1, 5, 10, 15, and 20 washing cycles. Values are mean ± standard deviation across *n* = 3 specimens. Face and reverse sides were measured separately for the knitted fabrics; Fabric 3 was evaluated in the orientation designated F3F.

Number of Washing Cycles	Fabric 1		Fabric 2		Fabric 3
	Face (Ω)	Reverse (Ω)	Face (Ω)	Reverse (Ω)	Face (Ω)
0	0.31 ± 0.05	0.34 ± 0.05	40.07 ± 3.75	1.37 ± 0.11	0.03 ± 0.00
1	0.33 ± 0.05	0.36 ± 0.05	41.00 ± 3.80	1.50 ± 0.12	0.03 ± 0.00
5	0.40 ± 0.06	0.48 ± 0.06	42.00 ± 3.90	1.75 ± 0.15	0.03 ± 0.01
10	0.50 ± 0.07	0.60 ± 0.07	41.50 ± 3.80	1.95 ± 0.18	0.03 ± 0.01
15	0.47 ± 0.06	0.58 ± 0.07	40.80 ± 3.70	1.90 ± 0.17	0.03 ± 0.01
20	0.52 ± 0.08	0.65 ± 0.08	40.20 ± 3.70	2.05 ± 0.20	0.03 ± 0.01

**Table 5:** Electrical resistance (Ω) measured under dry, acidic, alkaline, and neutral artificial sweat conditions for the face and reverse sides. For each of the three exposure media, values are the mean ± SD of nine repeated measurements (three positions × three readings) obtained from one independent specimen per fabric–surface–solution combination, so the SD reflects within-specimen measurement repeatability rather than specimen-to-specimen variability. The dry row reports the pre-immersion baseline of all three specimens of each fabric surface (27 readings); therefore, its SD also includes between-specimen variability. No inferential comparison between exposure conditions was performed.

Exposure Condition	Electrical Resistance (Ω)				
	F1F	F1R	F2F	F2R	F3F
Dry	0.32 ± 0.05	0.35 ± 0.05	40.50 ± 3.80	1.40 ± 0.12	0.03 ± 0.00
Acidic artificial perspiration	0.14 ± 0.02	0.17 ± 0.04	32.45 ± 7.31	1.23 ± 0.08	0.03 ± 0.00
Alkaline artificial perspiration	0.15 ± 0.03	0.17 ± 0.02	19.82 ± 7.17	1.39 ± 0.14	0.03 ± 0.00
Neutral water control	0.17 ± 0.02	0.20 ± 0.03	16.19 ± 3.49	1.45 ± 0.14	0.03 ± 0.00

**Table 6:** Reduced $R_{s}$–CPE parameters and fit quality for the electrode–phantom impedance spectra.

Electrode	$R_{s}$ (Ω), Median [IQR]	Q ($\mu S\cdot s^{\alpha }$), Median [IQR]	α, Median [IQR]	Complex NRMSE, RC/$R_{s}$–CPE (%)
Ag/AgCl comparison	299.5 [292.9–312.1]	1142.52 [963.77–1635.96]	0.636 [0.526–0.681]	1.58/0.15
F1F	150.3 [150.0–150.4]	1412.06 [1345.95–1444.08]	0.433 [0.432–0.434]	6.48/0.41
F1R	139.7 [136.0–145.6]	2730.43 [2609.10–2881.67]	0.283 [0.274–0.311]	5.88/0.73
F2F	Not resolved within 20–100 Hz	1.33 [1.31–1.35]	0.774 [0.772–0.775]	20.78/4.01
F2R	158.1 [156.5–159.5]	2281.75 [2147.58–2323.55]	0.475 [0.463–0.511]	3.48/0.28
F3F	Not resolved within 20–100 Hz	48.61 [46.50–49.87]	0.545 [0.544–0.548]	26.84/2.86

$R_{s}$, Q, and α are reported as medians [interquartile ranges] across the sixteen complete technical sweeps retained for each electrode, all acquired without changing the electrode–phantom placement. These sweeps are technical repetitions and do not represent independent specimens, independent placements, or repeated donning; therefore, the interquartile ranges characterize only within-placement technical repeatability. NRMSE values are the normalized complex error of the ideal RC and reduced $R_{s}$–CPE fits to the median complex spectrum, whereas $R_{s}$, Q, and α summarize the separate sweep-level fits. $R_{s}$ was reported as unresolved when its estimate remained at the lower fitting boundary within the available 20–100 Hz interval. Because Q has α-dependent units, it should not be interpreted as a conventional capacitance unless α=1 or it is compared directly across electrodes with different α.

**Table 7:** Temporal correlation coefficients between the textile and Ag/AgCl comparison signals during eyes-open and eyes-closed conditions. Correlations were computed per subject and averaged on the Fisher-z scale before back-transformation; values represent the back-transformed mean ± standard deviation across *n* = 9 subjects.

Condition	Pearson Correlation			
	F1F	F1R	F2R	F3F
Eyes-open	0.925 ± 0.067	0.878 ± 0.077	0.902 ± 0.032	0.877 ± 0.052
Eyes-closed	0.953 ± 0.034	0.910 ± 0.071	0.951 ± 0.036	0.940 ± 0.027

**Table 8:** Linear mixed-effects model on logit-transformed coherence, g ~ condition × band × configuration + (1|subject), *n* = 9 (288 estimates). Family-wise adjustment by the Holm procedure; the intercept is omitted. No gamma band was analyzed.

Effect	df1	df2	*F*	*p*	*p* (Holm)
Band	3	279	12.0	2.1 × 10^−7^	1.4 × 10^−6^
Configuration	3	279	1.57	0.196	0.784
Condition	1	279	5.07	0.025	0.151
Configuration × Condition	3	279	0.66	0.578	1.00
Configuration × Band	9	279	1.65	0.101	0.503
Condition × Band	3	279	1.16	0.327	0.982
Configuration × Condition × Band	9	279	0.12	0.999	1.00

## Data Availability

The data presented in this study are not publicly available due to privacy and ethical restrictions associated with human EEG recordings.

## Abbreviations

The following abbreviations are used in this manuscript:

ANOVA	Analysis of variance
BCI	Brain–computer interface
BIAS/DRL	Bias/driven-right-leg electrode
BIC	Bayesian information criterion
CCC	Lin’s concordance correlation coefficient
CI	Confidence interval
CPE	Constant-phase element
DSC	Differential scanning calorimetry
EC	Eyes-closed
ECG	Electrocardiography
EEG	Electroencephalography
EL	Elastane
EO	Eyes-open
FFT	Fast Fourier transform
GG	Greenhouse–Geisser (correction)
IEC	International Electrotechnical Commission
IIR	Infinite impulse response (filter)
IQR	Interquartile range
ISO	International Organization for Standardization
LCR	Inductance–capacitance–resistance meter
LoA	Limits of agreement
NRMSE	Normalized root-mean-square error
PA	Polyamide
PA6	Polyamide 6
PA66	Polyamide 66
PSD	Power spectral density
REF	Reference electrode
RMS	Root-mean square
RMSE	Root-mean-square error
RM-ANOVA	Repeated-measures analysis of variance
SD	Standard deviation
SEM	Scanning electron microscopy
TOST	Two one-sided tests (equivalence)
UNE	Una Norma Española (Spanish standard)
